# An Intelligent Synthetic Bacterium for Chronological Toxicant Detection, Biodegradation, and Its Subsequent Suicide

**DOI:** 10.1002/advs.202304318

**Published:** 2023-09-13

**Authors:** Huan Liu, Lige Zhang, Weiwei Wang, Haiyang Hu, Xingyu Ouyang, Ping Xu, Hongzhi Tang

**Affiliations:** ^1^ State Key Laboratory of Microbial Metabolism Joint International Research Laboratory of Metabolic and Developmental Sciences, and School of Life Sciences and Biotechnology Shanghai Jiao Tong University Shanghai P. R. China

**Keywords:** biosensors, chronological order, degradation, integrated system, long‐term stability, suicide switch

## Abstract

Modules, toolboxes, and synthetic biology systems may be designed to address environmental bioremediation. However, weak and decentralized functional modules require complex control. To address this issue, an integrated system for toxicant detection and biodegradation, and subsequent suicide in chronological order without exogenous inducers is constructed. Salicylic acid, a typical pollutant in industrial wastewater, is selected as an example to demonstrate this design. Biosensors are optimized by regulating the expression of receptors and reporters to get 2‐fold sensitivity and 6‐fold maximum output. Several stationary phase promoters are compared, and promoter P*_fic_* is chosen to express the degradation enzyme. Two concepts for suicide circuits are developed, with the toxin/antitoxin circuit showing potent lethality. The three modules are coupled in a stepwise manner. Detection and biodegradation, and suicide are sequentially completed with partial attenuation compared to pre‐integration, except for biodegradation, being improved by the replacements of ribosome binding site. Finally, a long‐term stability test reveals that the engineered strain maintained its function for ten generations. The study provides a novel concept for integrating and controlling functional modules that can accelerate the transition of synthetic biology from conceptual to practical applications.

## Graphical abstract

An integrated system for toxicant detection and biodegradation, and subsequent suicide in chronological order without exogenous inducers is constructed. The intelligent strain efficiently detects salicylic acid and produces red fluorescence after 6 h. Subsequently, salicylic acid is degraded into gentisic acid in the early stationary phase. Finally, the engineered strain autonomously activates the suicide system when salicylic acid disappears.

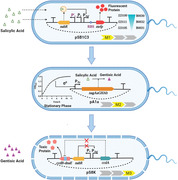

## 1 Introduction

Environmental pollution has an increasingly profound impact on national health and economic development, and synthetic biology has shed new light on solving this problem. Therefore, several synthetic modules, tools, and systems have been designed focusing on three main aspects: detection, degradation, and suicide system.^[^ ^1^ ^]^ Whole‐cell biosensors have attracted attention because of their low costs, high selectivity, and ease of manufacturing.^[^ ^2^ ^]^ Nucleic acid‐ and protein‐based biosensors are the two most common types that can regulate the expression of output signals by conformational alterations when binding to an input ligand;^[^ ^3^ ^]^ for instance, there are such sensors that include the guanidine‐bound *S. acidophilus* guanidine‐I riboswitch, ArsR for arsenic detection, MerR for mercury detection, and DmpR for detecting organophosphate pesticides containing phenolic groups.^[^ ^4^, ^5^, ^6^ ^]^ The analysis of catabolic pathways in natural strains facilitates the migration of functional genes to artificial cells that do not possess efficient or complete degradation abilities.^[^ ^5^, ^7^ ^]^ For example, an artificial consortium of three *E. coli* BL21(DE3) strains with synergistic functional modules was designed to completely degrade phenanthrene.^[^ ^8^ ^]^ A consortium comprised of an engineered *Escherichia coli* DH5α containing a gene cassette (*camA, camB, and camC*) that oxidizes hexachlorobenzene to pentachlorophenol and a natural pentachlorophenol degrader, *Sphingobium chlorophenolicum* ATCC 39723, was assembled for degradation of hexachlorobenzen.^[^ ^9^ ^]^ Restraining proliferation is one of the primary challenges faced by genetically modified microorganisms. There are many pioneering biocontainment strategies, including engineered prevention of self‐replication, auxotrophy, synthetic gene circuits, and integrated killing systems.^[^ ^10^, ^11^ ^]^

Salicylic acid (SA), a typical pollutant in industrial wastewater, was selected as the example compound. There are some reported SA biosensors, for example, Lux‐ and GFP‐based *Acinetobacter*, MarR‐P*_marO_* from *E. coli*, and TetR‐family repressor CmeR from the gastroenteric pathogen *Campylobacter jejuni*.^[^ ^12^, ^13^, ^14^ ^]^ SA is a key downstream node of the degradation of polycyclic aromatic hydrocarbons (PAHs), which benefited the construction of artificial degradation modules.^[^ ^7^, ^8^ ^]^ At present, there is no suicide circuit controlled by SA; thus, three independent modules (detection, biodegradation, and suicide) are not integrated.

However, weak and decentralized functional modules for detection, degradation, and biosafety require comprehensive control conditions, which hinder the ability of synthetic biology to solve environmental problems. First, a stable contaminant concentration is important for a biosensor to produce a reliable output signal, allowing similar dose‐response curves to analyze pollutant concentrations across different batches of experiments. On the one hand, this requires the strain to start degrading after the acquisition of biosensor signals; on the other hand, the time limit favors high degradation rates owing to the increase in biomass, like high‐density fermentation.^[^ ^15^ ^]^ It is necessary to kill engineered cells after completing the degradation of the target compounds; however, most strategies depend on exogenous inducers or physical conditions.^[^ ^11^ ^]^ Ultimately, the integrated system must be optimized to maintain all the functions of the original individual modules, and in environmental remediation, long‐term stability and robustness must be considered. Several researchers have attempted to achieve these objectives. For example, the 9‐kb naphthalene‐degrading gene *nahAD* was cloned into *Acinetobacter* ADPWH_lux, capable of responding to salicylate.^[^ ^16^ ^]^ An efficient Hg^2+^ adsorption strain with a biocontainment system was designed,^[^ ^17^ ^]^ and it achieved an Hg^2+^ adsorption efficiency of >95% with an escape rate of <10^−9^. To measure the long‐term stability and robustness of kill switches, cells containing suicide circuits were passaged for four days under survival conditions to periodically test the function of the circuits.^[^ ^10^ ^]^ However, there is still an urgent need for a synthetic biology system capable of autonomously and efficiently performing multiple integrated functions in chronological order without exogenous chemical inducers.

**Figure 1:**
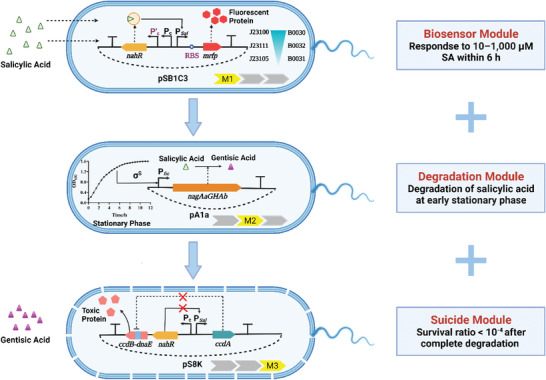
The working mechanism of an integrated engineered strain (created with BioRender.com). First, salicylic acid (SA) binds to NahR and activates the expression of mRFP when **Module 1** (**Biosensor**) is working. If the strain grows to the late exponential phase, the stationary phase promoter will begin transcription of salicylate 5‐hydroxylase (S5H), and SA will be degraded when **Module 2** (**Biodegradation**) is working. After complete depletion of SA, the suicide circuit will express a toxic protein to affect the growth of the strain when **Module 3** (**Lethality**) is working.

To address these challenges, we assembled a three‐module engineered strain that efficiently detected SA and produced red fluorescence after 6 h. Subsequently, SA was degraded into gentisic acid in the early stationary phase. Finally, the engineered strain autonomously activated the suicide system when SA disappeared. All tasks were completed sequentially using the engineered strain without intervention (**Figure** **1**). In order to optimize the independent modules, we constructed cross combinations of promoters‐ribosome binding sites (RBSs), collected a library of stationary‐phase promoters, and designed two suicide circuits. The attenuation in the integrated strain was ameliorated by replacement of RBSs and the long‐term stability was proved by continuous passage. Thus, our study sheds new light on functional normalization and timing control of synthetic biology modules used to treat environmental pollutants.

## 2 Results

### 2.1 Module 1: Biosensors for Salicylic Acid

**Figure 2:**
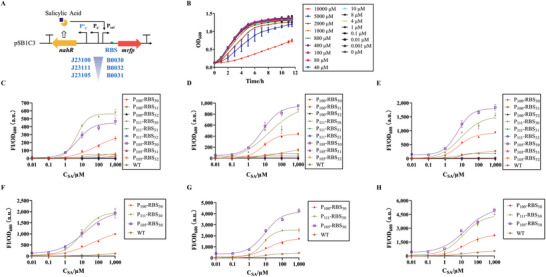
The principle and optimization of the biosensor for SA. A), NahR is a LysR‐type activator of *nah* and *sal* promoters responding to SA. We used promoters of different strengths to regulate the density of the receptor, and ribosome binding sites (RBSs) of different strengths to adjust the reporter expression. J231XX and B003X are registration numbers of promoters and RBSs in the iGEM registry. B), Growth curves of *E. coli* Top10 at different concentrations of SA. C–E), Dose‐Response curves of the biosensor with different optimized combinations in 96‐well plate, C–E are the results for 6, 8, and 10 h. WT is the wild‐type biosensor, and P_1XX_‐RBS_3X_ represents different optimized combinations (Promoter J231XX and RBS B003X). F–H), Dose‐Response curves of three candidate combinations in shake flasks, (F**–**H) are the results for 6, 8, and 10 h. Values are mean ± s.d. (*n* = 3 biologically independent samples).

NahR, a LysR‐type transcriptional activator of the *nah* and *sal* promoters,^[^ ^18^ ^]^ responds to salicylate and can be constructed as a biosensor to conveniently detect SA concentrations at a low cost. A wild‐type biosensor (WT, an unmodified sensor) was constructed using mRFP as the reporter (**Figure** **2**A); however, its low response and narrow dynamic and detection ranges limited its application (Figure 2F–H and **Table** **1**). Therefore, this sensor was optimized in two ways: regulating receptor density and reporter intensity. Promoters and ribosome binding sites (RBSs) of different intensities were selected from the iGEM registry (https://technology.igem.org/registry) to determine the optimal combination (Figure 2A). P_1XX_‐RBS_3X_ represents the biosensor optimized using each promoter (J231XX, registry number in iGEM) and RBS (B003X, registry number in iGEM) (Table 1 and Figure 2). To avoid the effect of SA on strain growth, a growth curve under gradient concentrations of SA was plotted (Figure 2B); 0.001–5000 µM SA did not inhibit cell growth. In the pre‐selection of the 96‐well plate, only three combinations showed noticeable improvements (Figure 2C–E). Compared with WT, their detection limits decreased to 0.1 mM and the response value increased from ≈50 to a maximum of 600 at 8 h. However, there were also several disadvantages, with leakage on P_105_‐RBS_30_ at 8 and 10 h and FI/OD_600 nm_ reaching saturation at a lower concentration.

**Table 1:** Parameters of biosensor performance with standard error of mean.

Sensor	Sensitivity	K_d_ [µM]	Dynamic range (FI/OD_600 nm_)	Inducer range [µM]	R^2^
WT (6 h)	0.37 (± 0.10)	MD	0–318.5	10–1000	0.9786
P_100_‐RBS_30_ (6 h)	0.52 (± 0.08)	42.0 (± 17.1)	6.9–1172.4	0.1–1000	0.9895
P_111_‐RBS_30_ (6 h)	0.82 (± 0.06)	10.0 (± 1.0)	42.3–1988.9	0.1–1000	0.9957
P_105_‐RBS_30_ (6 h)	0.58 (± 0.11)	15.0 (± 6.0)	107.3–2035.2	0.1–1000	0.9760
P*_fic_*‐TAT‐P_100_‐RBS_30_ (6 h)	0.59 (± 0.13)	85.2 (± 45.0)	9.0–306.4	3–1000	0.9657
P*_fic_*‐TAT‐P_111_‐RBS_30_ (6 h)	0.77 (± 0.14)	17.0 (± 4.1)	40.8–599.9	1–500	0.9590
P*_fic_*‐TAT‐P_105_‐RBS_30_ (6 h)	0.83 (± 0.12)	69.2 (± 15.0)	153.9–1402.2	3–1000	0.9772
P*_fic_*‐TAT‐P_100_‐RBS_35_ (6 h)	1.28 (± 0.17)	45.7 (± 5.4)	49.5–637.5	3–1000	0.9743
P*_fic_*‐TAT(2)‐P_100_‐RBS_35_‐G10 (6 h)	0.64 (± 0.12)	253.5 (± 151.05)	64.8–877.7	10–1000	0.9908
WT (8 h)	0.85 (± 0.16)	8.7 (± 2.0)	0–427.0	1–1000	0.9905
P_100_‐RBS_30_ (8 h)	0.71 (± 0.07)	12.6 (± 2.1)	2.3–1773.5	0.1–1000	0.9917
P_111_‐RBS_30_ (8 h)	1.25 (± 0.09)	6.8 (± 0.4)	107.5–2517.3	1–100	0.9972
P_105_‐RBS_30_ (8 h)	0.85 (± 0.10)	9.2 (± 1.3)	339.5–4180.8	1–1000	0.9905
P*_fic_*‐TAT‐P_100_‐RBS_30_ (8 h)	0.95 (± 0.18)	77.0 (± 17.5)	45.0–262.6	10–500	0.9651
P*_fic_*‐TAT‐P_111_‐RBS_30_ (8 h)	1.08 (± 0.12)	26.2 (± 3.7)	100.9–590.1	3–500	0.9762
P*_fic_*‐TAT‐P_105_‐RBS_30_ (8 h)	1.34 (± 0.19)	45.0 (± 5.0)	256.2–1241.2	6–500	0.9772
P*_fic_*‐TAT‐P_100_‐RBS_35_ (8 h)	1.40 (± 0.08)	154.6 (± 8.8)	179.7–2799.6	6–1000	0.9978
WT (10 h)	0.59 (± 0.12)	26.3 (± 12.3)	0–581.5	1–1000	0.9682
P_100_‐RBS_30_ (10 h)	0.73 (± 0.09)	17.2 (± 3.3)	2.9–2327.0	0.1–1000	0.9889
P_111_‐RBS_30_ (10 h)	0.74 (± 0.11)	13.2 (± 3.0)	78.5–4434.4	0.1–1000	0.9843
P_105_‐RBS_30_ (10 h)	0.62 (± 0.10)	17.4 (± 5.7)	348.0–5203.0	0.1–1000	0.9793
P*_fic_*‐TAT‐P_100_‐RBS_30_ (10 h)	1.33 (± 0.27)	87.5 (± 11.4)	169.1–2366.2	10–500	0.9696
P*_fic_*‐TAT‐P_111_‐RBS_30_ (10 h)	1.47 (± 0.15)	122.4 (± 9.7)	69.3–1150.7	10–500	0.9932
P*_fic_*‐TAT‐P_105_‐RBS_30_ (10 h)	1.14 (± 0.16)	113.3 (± 17.0)	412.3–4323.0	10–1000	0.9844
P*_fic_*‐TAT‐P_100_‐RBS_35_ (10 h)	1.06 (± 0.08)	217.2 (± 27.8)	162.2–2960.4	6–1000	0.9960

Sensitivity: Hill slope of fitted data. Kd: concentration of SA to achieve half predicted maximal fluorescence intensity (FI)/OD_600 nm_ value. Dynamic range: minimal and maximal predicted FI/OD_600 nm_ values. Inducer range: the range of SA which can be detected by the biosensor determined by experiments. WT is the unmodified biosensor. P_1XX_‐RBS_3X_ represents different optimized combinations of promoters and RBSs for biosensors. P_*fic*_‐TAT‐ P_1XX_‐RBS_3X_ indicates that the triple‐plasmid transformant contains pSB1C3‐1XX‐3X (optimized biosensor), pA1a‐P_*fic*_‐*nagAaGHAb* (biodegradation) and pS8K‐toxin/antitoxin (suicide circuit). All parameters were obtained by analysis of data measured in shake flasks. MD means meaningless data. Values are mean ± s.d. (*n* = 3 biologically independent samples).

Owing to oxygen and mass transfer limitations in the 96‐well plate, their functions were tested in shake flasks. Compared to the WT at 6 h (Figure 2F and Table 1), the sensitivity of each promoter increased in the order of middle (J23111), low (J23105), and high (J23100) promoter intensity, which was consistent with the order of decrease in half‐maximal activation concentration K_d_. Their dynamic range was extended, especially the maximum output, which changed from 318.5 to 2035.2, while the order of the maximum outputs was opposite of the promoter intensities. Optimized sensors could detect 0.1 µM SA, two orders of magnitude lower than WT. Over time, the sensors became more sensitive, and the highest sensitivity was obtained at 8 h (1.27 of P_111_‐RBS_30_, Figure 2G,H and Table 1). However, the K_d_ values of P_111_‐RBS_30_ and P_105_‐RBS_30_ at 10 h were higher than those at 6 h. The dynamic range was widened due to the continuous differential expression of mRFP, even though leaky expression became more significant. Except for P_111_‐RBS_30_ and P_105_‐RBS_30_ at 8 h, the detection range was 0.1–1000 µM SA for each combination at all periods. Meanwhile, the WT improved on some key parameters of the biosensor; for example, the maximum output of WT changed from 318.5 to 581.5, which still lagged behind the optimized groups. To sum up, our optimized biosensor (P_111_‐RBS_30_ at 6 h) showed an ≈2‐fold increase in sensitivity, a 6‐fold increase in maximum output, and a two‐orders‐of‐magnitude decrease in detection limits compared to the WT.

### 2.2 Module 2: Stationary‐Phase Biodegradation of Salicylic Acid

**Figure 3:**
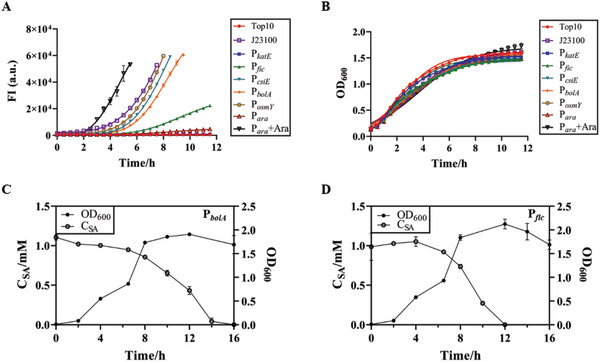
Selection of stationary phase promoters and characterization of the biodegradation circuit. A), Fluorescence intensity curves were driven by different stationary phase promoters. “Top 10″ means a wild‐type strain without mRFP, “J23100” and “P*_ara_*” represent the expression of mRFP by a constitutive promoter J23100 and an inducible promoter P*_ara_*, respectively. “Ara” is arabinose. B), Growth curves of the strains containing different stationary phase promoters. C–D), Growth and degradation curves of a strain containing S5H driven by P*_bolA_* or P*_fic_* promoter. Values are mean ± s.d. (*n* = 3 biologically independent samples).

Stationary phase promoters respond to starvation and cellular stress by transcribing downstream genes via RNA polymerase containing the σ^S^ subunit (a product of the *ropS* gene).^[^ ^19^ ^]^ In the time dimension, gene expression was activated when the strains grew to the stationary phase in rich media. Five genes (*bolA*, *csiE*, *katE*, *fic*, *osmY*) were previously recognized as *rpoS* dependent; therefore, the corresponding five promoters were amplified from the *E. coli* BL21(DE3) genome using the primers described in a previous study,^[^ ^20^ ^]^ and mRFP was introduced as a reporter to characterize these promoters and select the best two. Three criteria were established: 1) cell growth was not affected; 2) the start of transcription was strict, and the natural stationary phase promoters were induced early in the late exponential phase;^[^ ^20^ ^]^ 3) it had a detectable output intensity. P*_katE_* showed no activity, whereas P*_osmY_* and P*_csiE_* turned on much earlier than the late exponential phase (>2 h, **Figure** **3**A,B), requiring RT‐qPCR to determine whether it was a leaky expression or its features. P*_bolA_* and P*_fic_*, which met the above criteria were characterized by salicylate 5‐hydroxylase (S5H); P*_bolA_* was ≈3‐fold stronger than P*_fic_*. The growth‐degradation curves at 1 mM SA were plotted against the plasmid vector pA1a. The engineered strains grew to the late exponential phase at ≈8 h, and SA began to be degraded after 6 h (Figure 3C,D). The strain with P*_bolA_* completely degraded SA in 16 h, and another strain with P*_fic_* within 12 h was opposite to the intensities characterized by mRFP. Stronger promoters may not result in higher enzyme activities such as feedback regulation or protein misfolding.^[^ ^21^ ^]^ Therefore, we regarded P*_fic_* as the optimal stationary phase promoter for constructing the degradation circuit.

### 2.3 Module 3: Suicide Circuits

**Figure 4:**
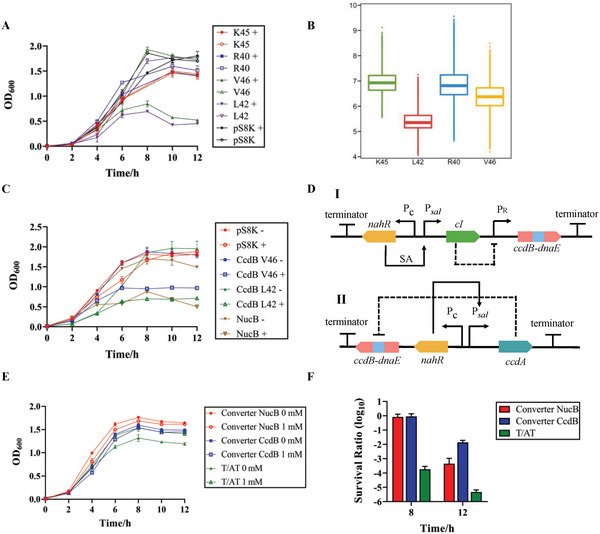
Selection of toxic proteins and characterization of two suicide circuits. A and C), Growth curves of strains containing different toxic proteins, CcdB with different split sites and all functional toxic proteins are driven by an inducible promoter. “+” represents induction by arabinose, and “pS8K” is the strain with an empty vector. B), The box plot of distances between the key residues C_α_‐C_α_ for the complexes of CcdB and DnaE with different split sites. D), Two designs of suicide circuits. Circuit I is based on a “gene converter” by repressor CI which causes SA to inhibit the expression of CcdB. Circuit II is based on the toxin/antitoxin pair, where the expression of CcdA is activated by SA, and the expression of CcdB is constitutive. E and F), Growth curves, and survival ratios of strains containing two kinds of suicide circuits. 0 and 1 mM indicate different SA concentrations. Values are mean ± s.d. (*n* = 3 biologically independent samples).

Several toxic proteins were selected to test their functions, including CcdB, NucB, HokD, MazF, Gp2, RelK, and ProE (Table S2, Supporting Information). When the expression of toxic proteins was induced with arabinose, strains containing HokD, MazF, RelK, ProE, or Gp2 did not show any growth differences compared with the non‐induced group (Figure S2, Supporting Information). Unfortunately, transformants positive for CcdB were not obtained because basal CcdB expression was sufficient to kill the cells; to avoid this, intein was used to decrease the toxicity of intact CcdB, which was embedded in the host protein and autocatalytically excised during protein splicing before producing the mature protein.^[^ ^22^ ^]^ NucB, CcdB‐L42, and CcdB‐V46 inhibited growth after 4 h of induction, and the lethal effect of CcdB‐L42 was greater than that of V46 (**Figure** **4**A,C).

Molecular dynamics (MD) simulations were used to verify whether the experimental results matched the selection of the split sites. According to the structural prediction using AlphaFold2.3.1, the complexes of CcdB and DnaE did not change their original structures (Figure S3A, Supporting Information). The distances between key residues C_α_‐C_α_ were sampled during MD simulations ( Figure S3B,C, Supporting Information), which suggested that the split CcdB at Arg42 was easiest to be restored due to the smallest distance between two key residues (Figure 4A,B). The order of these distances was: L42 > V46 > R40 > K45, which was consistent with the growth curves of strains containing the split CcdB (Figure 4A,C).

The suicide circuits were designed using two concepts (Figure 4D). The first one is based on a “gene converter” using NucB and CcdB‐L42, respectively, where the CI repressor binds to the *cI* regulator and blocks the expression of downstream genes.^[^ ^23^ ^]^ Therefore, SA activates CI expression, which suppresses the expression of the following toxic genes; the other was constructed using a pair of toxin/antitoxin (T/AT) proteins: CcdB‐L42 (toxin) and CcdA (antitoxin).^[^ ^24^ ^]^ CcdA could prevent CcdB from interacting with DNA gyrase by binding to CcdB.^[^ ^25^ ^]^ Therefore, when SA is present, the strain survives because the expression of CcdA is activated by NahR using SA as an inducer to deactivate CcdB. In contrast, CcdB causes cell death if SA is consumed, resulting in cessation of CcdA expression and the accumulation of CcdB. When all suicide circuits were compared in cell growth curves, only the T/AT circuit showed a negative effect (Figure 4E). Colony‐forming unit (CFU) is a common method of characterizing the lethal or survival ratio. In the T/AT circuit, the cells under survival conditions were 10^3^ times more than those under dead conditions.

In contrast, the others showed no difference at 8 h (Figure 4F). After 4 h, the dead cells increased due to the accumulation of toxic proteins. The T/AT circuit maintained the most powerful lethal ability, the survival ratio of which decreased to 10^−5^. According to the CFU results, NucB was better at inducing cell death than CcdB‐L42 in the converter circuit. Therefore, the T/AT circuit was used as a suicide system for engineered strain.

### 2.4 Integration of Three Modules and Optimization of Intelligent Strains

**Figure 5:**
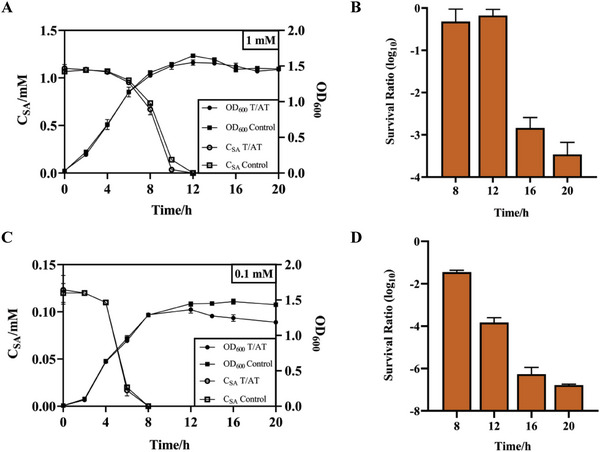
Characteristics of double‐plasmid transformants containing biodegradation and suicide circuits. A,B), Growth curves, degradation curves and survival ratios of double‐plasmid transformants containing biodegradation and suicide circuit at 1 mM SA. Control is the double‐plasmid transformant containing the biodegradation module and pS8K vector. C,D), The above characterization at 0.1 mM SA. CFU for 20 h was measured owing to the accumulation of CcdA. Values are mean ± s.d. (*n* = 3 biologically independent samples).

To ensure that the strain sequentially completed sensing, degradation, and suicide, the three modules were integrated into one strain, and their functions were tested. First, we obtained a double‐plasmid transformant with degradation and suicide. The characteristics of the stationary phase promoter did not change (**Figure** **5**A), i.e., degradation started and ended simultaneously as in the single transformant. Next, CFU was measured for 20 h because of the accumulation of CcdA. Differences in growth curves did not appear, and the minimum survival ratio decreased to 10^−3^, similar to the data obtained when the suicide circuit was tested separately at 8 h (Figures 4F and 5A,B). The general trend of the survival ratio was that it was maintained at a high level until SA was degraded entirely and then decreased to a lower stage.

In many cases, the SA concentration might be lower than 0.1 mM;^[^ ^26^ ^]^ therefore, we tested the strain at 0.1 mM SA. SA was degraded from 4 h onward and was undetectable at 8 h (Figure 5C). This was because the leaky expression of S5H resulted in the significant degradation of SA at low concentrations. As a result, growth was inhibited, and the survival ratio was lower than that at 1 mM SA (Figure 5D). However, the survival ratio trend remained consistent with that of the former; the main reason for this phenomenon was the low expression of CcdA. mRFP was introduced into the suicide circuit to prove the low expression of CcdA in double‐plasmid transformant under 0.1 mM SA; the values of FI/OD_600 nm_ were lower than those under 1 mM SA at 8–12 h, SA was completely consumed after 12 h (Figure S5, Supporting Information).

**Figure 6:**
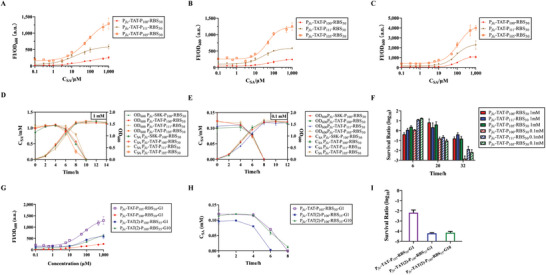
Characterization and optimization of triple‐plasmid transformants containing the biosensors, biodegradation module, and suicide circuit. A–C), Dose‐Response curves of the three best candidates among the triple‐plasmid transformants with different optimized biosensors in shake flasks. (A–C) are the results for 6, 8, and 10 h. P*_fic_*‐TAT‐P_1XX_‐RBS_3X_ indicates that the triple‐plasmid transformant contains pSB1C3‐1XX‐3X (optimized biosensor), pA1a‐P*_fic_*‐*nagAaGHAb* (biodegradation module) and pS8K‐toxin/antitoxin (suicide circuit), WT is the wild‐type biosensor. D–F), Growth curves, degradation curves, and survival ratios of the triple‐plasmid transformant containing the biosensor, biodegradation module, and suicide circuit at 1 mM and 0.1 mM SA. P*_fic_*‐S8K‐100‐30 is the triple‐plasmid transformant containing the biosensor, biodegradation module, and pS8K vector as a control. G–I), Dose‐Response curves, degradation curves, and survival ratios (at 0.1 mM SA) of the triple‐plasmid transformant containing biodegradation modules, optimized biosensor and suicide circuit in the first and tenth generation. CFU for 32 h was measured because the relative expression of CcdA and CcdB was changed by NahR of two circuits. Values are mean ± s.d. (*n* = 3 biologically independent samples.

The biosensor was introduced into a double‐plasmid transformant. This triple‐plasmid transformant responded to SA in the first few hours and degraded SA when the cells reached the late exponential phase. Finally, the suicide circuit inhibits cell growth in the absence of SA (Figure 1). During pre‐selection in a 96‐wells plate, the triple‐plasmid combinations showed the same ability to detect SA as single transformants (Figure S6A–C, Supporting Information). Similarly, we scaled up this experiment to shake the flasks and set up more detailed SA concentrations (**Figure** **6**A–C). As a result, the inducer range narrowed and K_d_ increased from 6 to 10 h. Therefore, we decided 6 h was the optimal time to read the sensor data (Table 1). The sensitivity, dynamic range, and inducer range of P*_fic_*‐TAT‐P_105_‐RBS_30_ (the triple‐plasmid transformant containing pA1a‐P*_fic_*‐*nagAaGHAb*, pSB1C3‐105‐30, and pS8K‐toxin/antitoxin) were the best among P*_fic_*‐TAT‐P_100_‐RBS_30_, P*_fic_*‐TAT‐P_111_‐RBS_30_, and P*_fic_*‐TAT‐P_105_‐RBS_30_, although its K_d_ was slightly higher than that of P*_fic_*‐TAT‐P_111_‐RBS_30_ at 6 h.

Degradation and lethality were tested at different SA concentrations (Figure 6D–F). SA (1 mM) was degraded faster than the double‐plasmid transformant, with visible degradation from 4 to 6 h, but 0.1 mM SA was degraded the same as before. Unfortunately, no inhibition was observed in the growth curves at either concentration. CFU for 32 h was measured because NahR altered the two circuits’ relative expression of CcdA and CcdB. The lethal effect also significantly decreased, with an ≈10‐fold difference in CFU in the surviving group compared with the lethal group at 1 mM SA until 32 h. Even though all survival ratios of the different groups at 0.1 mM SA decreased from 20 h, the minimum survival ratio at 32 h was only two orders of magnitude lower than that at 1 mM SA. Based on a t‐test, there was no significant difference between the survival ratio of P*_fic_*‐TAT‐P_105_‐RBS_30_ and P*_fic_*‐TAT‐P_111_‐RBS_30_ cells treated with 0.1 mM SA for 32 h, which was only one order of magnitude higher than that of P*_fic_*‐TAT‐P_100_‐RBS_30_.

To ameliorate the attenuated biosensor and suicide circuit, the CcdA in the suicide circuit and mRFP in the low‐leakage biosensor were down‐ and up‐regulated by replacement of RBSs, respectively. We observed that a stronger promoter for the regulator caused lower leakage and a narrow dynamic range, and a stronger RBS for the reporter resulted in higher maximum output (Figure 6A–C). It is also clear that the accumulation of CcdA is the key to lethality. The optimized P*_fic_*‐TAT(2)‐P_100_‐RBS_35_ exhibited the same ability to degrade SA, lower leak expression of the biosensor, and two orders of magnitude lower survival ratio than that of P*_fic_*‐TAT‐P_105_‐RBS_30_. P*_fic_*‐TAT(2)‐P_100_‐RBS_35_ did not perform well in sensitivity, dynamic range, and detection range of the biosensor as P*_fic_*‐TAT‐P_105_‐RBS_30_ (Figure 6G–I, Table 1; Figure S6G, Supporting Information). However, when this optimized biosensor (P_100_‐RBS_35_) was transformed to the unmodified double‐plasmid transformant, P*_fic_*‐TAT‐P_100_‐RBS_35_ gave different biosensor characteristics, which were similar to those of P*_fic_*‐TAT‐P_105_‐RBS_30_ except for lower leakage (Figure S6D–F, Supporting Information). A long‐term stability test suggested that P*_fic_*‐TAT(2)‐P_100_‐RBS_35_ maintained its original dose‐response curve, degradation rate, and survival ratio after 10 generations (Figure 6G–I).

## 3 Discussion

Addressing environmental bioremediation needs focusing on three aspects: toxicant detection, biodegradation, and biosafety; however, weak and decentralized functional modules need complex control conditions and enhancement of function when using synthetic biology. To address this issue, an integrated system for toxicant detection and biodegradation, and subsequent suicide in chronological order without exogenous inducers was investigated in environmental microbiology, which gives GMOs (Genetically Modified Organism) versatility under autonomous control and ensures biosafety.

First, we optimized each of the three integrated modules. For detection, two common but effective methods were used to optimize our biosensor:1) regulating the density of the receptor and 2) modulating the intensity of the reporter, which has already been applied to valerolactam and caprolactam biosensor.^[^ ^27^ ^]^ The former strategy changed the sensitivity and detection range, whereas the latter was used to increase the output signal (Figure 2C–H, Table 1). Although an extremely weak promoter can cause high basal expression or low output signals, the strongest promoter does not produce the best biosensor.^[^ ^28^ ^]^ Compared with our optimal SA biosensor, Lux‐ and GFP‐based *Acinetobacter* exhibited narrower salicylate detection ranges of 1–100 and 10–100 µM, respectively.^[^ ^12^ ^]^ The wild‐type MarR‐P*_marO_* sensor in *E. coli* required a response time of 24 h, which was six times longer than ours.^[^ ^13^ ^]^ CmeR in *E. coli* could only detect 100–1000 µM salicylate in 20–24 h.^[^ ^14^ ^]^ There are many other methods for optimizing the biosensors, such as promoter and RBS engineering, replication origin engineering, regulator protein engineering, and cascaded amplifiers.^[^ ^28^, ^29^, ^30^, ^31^ ^]^ We assessed how time affected biosensor properties and found that sufficient time was required to produce responding signals and reach the optimal state; however, excessive time limited the application of biosensors.^[^ ^3^ ^]^ Computer‐assisted tuning approaches such as deep learning and machine learning can predict the performance of optimized biosensors.^[^ ^32^ ^]^

In order to express the degradation enzyme at a specific time for sufficient accumulation of fluorescence, we collected several common stationary phase promoters for the degradation module and characterized them using mRFP, focusing on their initiation times and intensities. Natural environmental bacteria are often subject to nutritional restrictions or environmental stress, under which some genes are expressed during the stationary phase; therefore, we can use stationary‐phase promoters to control gene expression in such strains by building a library of stationary‐phase promoters with different strength and transcription start time using transcriptome.^[^ ^33^ ^]^ Concerning metabolic regulation, stronger promoters do not result in faster degradation. Therefore, it is necessary to assemble a stationary phase promoter with the appropriate strength and induction time for a specific enzyme; this can be achieved by *de novo* synthesis and promoter engineering.^[^ ^34^ ^]^

In the suicide circuit, for an activator‐based transcription factor, repressor CI or T/AT pair was used to control the function of the toxic protein. Many toxic proteins, such as CcdB, are difficult to be constructed in circuits because of their powerful functions. Splitting these proteins can decrease the toxicity of leaked expression and maintain their original functions.^[^ ^35^ ^]^ However, the selection of the optimal split site limits its application. To reduce the scope of selection, existing databases and mathematical simulation tools can be used to predict the effects of split sites (Figure 4B; Figure S3, Supporting Information).^[^ ^36^, ^37^ ^]^ The lethality of “gene converter” was weaker because the CcdB expression could not be inhibited by the survival signal (1 mM SA) (Figure S4, Supporting Information), and the intensity of the P_R_ promoter was not sufficiently high. The performance of the T/AT circuit is closely related to the expression of antitoxin and the relative contents of the toxin and antitoxin.^[^ ^38^ ^]^

Moreover, we coupled the modules into one strain step‐by‐step, focusing on the effectiveness of chronological control and the integrity of the module function. When degradation and the suicide circuit were first combined, the survival ratio trend changed with the SA concentration, which could be caused by variations in CcdA expression. A delay between the completion of degradation and the lowest survival ratio occurred, highlighting the benefits of using proteases, a riboswitch‐integrase combined platform, or other means to accelerate the switching of regulatory proteins on and off.^[^ ^39^, ^40^, ^41^ ^]^ Each module was disturbed at a different level in the integrated system, which contained all three modules. Due to the degradation of SA over time, the biosensor characteristics changed more than those of the single transformant. Undoubtedly, the response time of the biosensor must be modulated by altering the growth medium or adding a hydrolysis label.^[^ ^42^ ^]^ After detection, the start and end time of degradation at 1 mM SA were 2 h earlier because the stationary phase promoter was deeply influenced by growth stress,^[^ ^15^ ^]^ and the three plasmids might introduce an extra metabolic burden. Unfortunately, both the biosensor and the suicide circuit have deteriorated because it is difficult to balance NahR expression in these two genetic circuits to avoid crosstalk between identical parts, or because the metabolic burden affects the function of each module. To summarize the changes in biosensors with different promoters and RBSs, a wider dynamic range, higher sensitivity, and lower leakage can be achieved by further strengthening the RBS of the reporter in P*_fic_*‐TAT(2)‐P_100_‐RBS_35_.

Regarding the suicide circuit, decreasing the expression of CcdA and increasing the expression of CcdB are regarded as effective means of increasing lethality; however, high lethality may affect the cellular activity and functional circuits. Like metabolic engineering, computer‐assisted design can accurately regulate the expression intensity of a specific protein or replace the same part with another functional regulatory element, such as a riboswitch.^[^ ^13^, ^41^, ^43^ ^]^ In addition, to make the circuits genetically stable without hindering the host, they can be introduced into highly insulated genomic landing pads.^[^ ^44^ ^]^ ADPWH_Nah combines detection and degradation; however, its detection range is limited by the weak activity of the degradation cluster.^[^ ^16^ ^]^ The biodegradation and biosafety modules were integrated into the BL21(DE3) AI‐GOS strain. However, an exogenous inducer is required to activate the killing circuit.^[^ ^45^ ^]^ Our engineered strain maintained its original function for ten generations, indicating the stability of this system and its potential for practical applications. Furthermore, it is necessary to integrate all the circuits into the genome to prevent plasmid loss and reduce antibiotic use; this is the first time three functional modules (biosensor, biodegradation, and biosafety) have been integrated into one strain to efficiently complete their corresponding tasks chronologically without any exogenous inducers.

In conclusion, we designed an integrated engineered strain that could perform sensing, degradation, and suicide in chronological order without any exogenous inducers (Figure 1). This strain (P*_fic_*‐TAT(2)‐P_100_‐RBS_35_) responded to 10–1000 µM SA within 6 h. Upon reaching the late exponential phase, the stationary phase promoter began the transcription of *nagAaGHAb* to degrade SA. Finally, the engineered strains killed themselves without SA, thereby ensuring biosafety. Moreover, the integrated strain exhibited long‐term stability and maintained its function for ten generations. This study optimizes each module to make it more powerful. It regulates the integrated system using logic gates and chronologically controlled parts, which solves the challenge of decentralized and inefficient functional modules.

## 4 Experimental Section

### Strains, Plasmids, Chemicals, and Growth Conditions

All plasmid cloning and characterization of the engineered genetic circuits were performed in *E. coli* Top10. Four plasmid backbones with different copy numbers, pS8K (low copy number), pA1a (middle), pSB1C3 and J61002 (high), were used, while constructed plasmid derivatives were listed in Table S1 (Supporting Information). All strains were cultured in Luria‐Bertani (LB) medium containing 10 g L^−1^ tryptone, 10 g L^−1^ NaCl, and 5 g L^−1^ yeast extract with appropriate antibiotics. Generally, the concentration of ampicillin, kanamycin, and chloramphenicol were 100, 50, and 25 mg L^−1^, respectively. However, the concentrations of antibiotics were halved in the experiment with the triple‐plasmid transformant. The concentrations of arabinose (Ara) and salicylic acid (SA) were 1 mol L^−1^ and 100 mmol L^−1^, respectively. Antibiotics, Ara, and SA were dissolved in ddH_2_O and filtered using 0.22 µm filters (Sango Biotech., F513161‐0001).

All engineered strains were first inoculated from individual colonies on LB solid plates to an appropriate volume of LB liquid medium, and cultured overnight at 37 °C with shaking (200 r.p.m.). For characterization, the seed cultures were then diluted 100‐fold into a fresh LB liquid medium under the same culture conditions.

### Genetic Circuits Construction and Transformation

All information for the genetic parts was listed in Table S2 (Supporting Information), detailed plasmid maps were shown in Figure S1 (Supporting Information), and primers were summarized in Table S3 (Supporting Information). Standard polymerase chain reaction (PCR) amplification and Gibson assembly were used to construct the genetic circuits and plasmids (Vazyme, C112). *De novo* synthesized genes were purchased from BGI, China. The plasmids were transformed into *E. coli* Top10 following standard protocols, and the resulting engineered strains were confirmed by Sanger sequencing (BGI).

### Characterization of Biosensors

NahR (regulator), mRFP (reporter), and P*_sal_* (cognate promoter of NahR) were used to design the biosensor. Constitutive promoters and ribosome binding sites (RBSs) with different strengths were introduced into the circuit by PCR. Constitutive promoters were from Anderson promoter collection, and ribosome binding sites were based on Ron Weiss thesis, which were both suitable for general protein expression in *E. coli* and likely other prokaryotes (http://parts.igem.org/). The seed cultures were diluted into an LB liquid medium containing gradient concentrations of SA, after which 200 µL of diluted culture was added to 96‐well plate for incubation to select the optimal combination of promoter and RBS (microporous plate oscillator, MBR‐420FL, Taitec). Samples were obtained at 6, 8, and 10 h. The fluorescence intensity (FI) of mRFP was measured by Tecan Spark fluorometry (583 ± 10 nm for excitation, 607 ± 10 nm for emission, gain = 120). At the same time, the optical density (OD_600 nm_) was read to represent cell density. The medium background of FI and OD_600 nm_ was determined by blank wells within fresh LB liquid medium and subtracted from the experiment groups. Data were processed by GraphPad Prism, and the dose‐response curve was fitted using the Sigmoidal, 4PL model. Candidates for the best combination were scaled up in a 250 mL flask containing 50 mL of medium. At least three experimental replicates were implemented for each experiment unless otherwise indicated.

### Characterization of Biodegradation

Stationary phase promoters, amplified from *E. coli* BL21(DE3) by PCR,^[^ ^20^ ^]^ were designed to express the salicylate 5‐hydroxylase (S5H). The strains with different stationary phase promoters and *mrfp* were cultured in 96‐well plates to compare their activities. FI and OD_600 nm_ were read at 0.5 h intervals until 12 h. For the best two, *mrfp* was replaced by *nagAaGHAb* and constructed in the pA1a, strains were scaled up in 250 mL flasks containing 100 mL LB and 1 mM SA. Samples were obtained every 2 h until SA was completely consumed. The OD_600 nm_ was measured as described above and the concentration of SA was detected by high‐performance liquid chromatography (HPLC) (Agilent Technologies 1200 series) with an Agilent Eclipse XDB‐C18 column (5 µm, 4.6 × 150 mm). The HPLC parameters were as follows: flow rate 0.5 ml min^−1^, flow phase 50% methanol and 50% deionized water with 0.1% (vol/vol) formic acid, column temperature 30 °C, detection wavelength 298 nm, and stop time 15 min.

### Characterization of Suicide Systems

Several toxic proteins with different mechanisms were first compared first in 50 mL flasks containing 10 mL LB. OD_600 nm_ was measured by sampling every 2 h until 12 h, and incubation was induced with a final concentration of 10 mM Ara at 2 h. The split sites of CcdB were determined using the methods of the iGEM project (2019.igem.org/Team:DUT_China_B), except that L42 was used in a previous study.^[^ ^22^ ^]^

The protein‐protein complex structure of truncated CcdB and DnaE was predicted by multimer module of AlphaFold2.3.1,^[^ ^46^ ^]^ the input amino acids sequences for prediction was shown in Table S3 (Supporting Information). The highest scoring complex predicted by AlphaFold2.3.1 was used as the initial structure of MD simulation, topology files including connection relation and coordination in ff14SB force field of complex were generated by tleap module in Amber20.^[^ ^47^, ^48^ ^]^ A box of TIP3P water with the thickness of the external water layer exceeding 10 Å of the protein was added to the whole complex to simulate the environment of the protein in solution, using sodium ions to keep the system charge neutralization. In the simulation process, the energy of the system was minimized by the 1000 steps steepest descent algorithm, and then 9000 steps were carried out by conjugate gradient minimization to complete the energy minimization of the system. The system gradually heated up from 0K to 300K through 25000 iterations, maintaining a constant volume throughout heating. Keeping 300k, the system was stabilized for 200 ps for equilibration in the NPT ensemble, and the SHAKE algorithm in its matrix form was used to fix bonds and angles involving hydrogen atoms. In the MD simulation sampling, different random numbers were selected for each simulation, and three trajectories were sampled in parallel for each system. Each trajectory includes structural information per 2 ps, with a total duration of 50 ns. Root mean square deviation analysis (RMSD) and distance analysis were analyzed by CppTraj in AmberTool21.^[^ ^49^ ^]^ PyMOL was applied to complete the structure visualization.^[^ ^50^ ^]^

Two toxic proteins were used to design suicide circuits by two different concepts—CcdB and NucB for gene converter, respectively, and CcdB for toxin/antitoxin pair. The engineered strains were cultured under survival conditions (containing SA at a final concentration of 1 mM) and dead conditions (without SA). To characterize the lethal efficiency, growth curves and survival ratios were measured in 250 mL flasks containing 50 mL LB. Survival ratios were calculated based on CFU using the following formula:

(1) $$\mathrm{Survival}\;\mathrm{ratio}=log_{10}\left(\frac{CFU\;of\;dead\;condition}{CFU\;of\;survival\;condition}\right)$$

CFU was the number of single colonies on the agar plate, which was more accurate when this number was between 30 and 300. Samples were serially diluted to a proper concentration, and 200 µL of the diluent was spread on the LB solid plate, which was incubated upside down at 37 °C overnight. To measure the expression of toxic protein in the “gene converter” circuit at the “off” state, *mrfp* was introduced into different circuit positions (Figure S4A, Supporting Information), and FI was measured as described above.

### Transformation and Characterization of Multiple‐Plasmid Strains

New plasmids were introduced into the engineered strain that already contained one or two plasmids using standard electrotransformation protocols. For the double‐plasmid transformant containing biodegradation and suicide system modules, it was cultured in a 250 mL flask containing 50 mL LB at a final concentration of 1 mM and 0.1 mM SA, respectively. Samples were obtained to measure the OD_600 nm_, concentrations of SA and survival ratio for 20 h. To prove that different concentrations of salicylic acid induced the differential expression of *ccdA*, *mrfp* was introduced to the rear of the *ccdA* in the toxin/antitoxin circuit which was transformed into the strain containing biodegradation module, and FI/OD_600 nm_ was read at 8, 10, and 12 h.

Characterization of triple‐plasmid transformant was the same as the double one for 32 h, expect for an extra biosensor parameter, FI. Weaker RBS for *ccdA* and stronger RBS for *mrfp* were replace into the suicide circuit and biosensor by PCR, and characterization of the optimized triple‐plasmid transformant was performed as above.

The triple‐plasmid transformant was passed 10 generations in the survival condition (1 mM SA), and the dose‐response curve, degradation rate, and survival ratios were tested at the first and tenth generation under 0.1 mM SA (the latter two parameters) to prove its long‐term stability.

### Statistical Analysis

Statistical analysis was performed by GraphPad Prism 8.0. Values were mean ± s.d. (*n* = 3 biologically independent samples). Significance analysis between the two data was conducted by t‐test using SPSS. The dose‐response curve was fitted using the Sigmoidal, 4PL model in GraphPad Prism 8.0.

## Conflict of Interest

The author declare no conflict of interest.

## Author Contributions

H.L. and L.Z. contributed equally to this work. H.L., L.Z., X.O., and H.T. outset and designed experiments. H.L. and L.Z. performed experiments. H.L., L.Z., X.O., H.H., W.W., and H.T. analyzed the data. H.T. and P.X. received projects, contributed reagents and materials. H.L., L.Z., and H.T. wrote the paper. All Authors importantly discussed and revised the manuscript. All Authors commented on the manuscript before submission. All authors read and approved the final manuscript.

## Supporting information

Supporting Information

## Data Availability

The data that support the findings of this study are available from the corresponding author upon reasonable request.
