# Supplementary material for: An Intelligent Synthetic Bacterium for Chronological Toxicant Detection, Biodegradation, and Its Subsequent Suicide
Source: Adv Sci (Weinh). 2023 Sep 13;10(31):2304318. doi: 10.1002/advs.202304318 (PMC10625131; doi:10.1002/advs.202304318)
Supplement: Supplementary file 1 — Supporting Information [file ADVS-10-2304318-s001.pdf]

ADVANCED SCIENCE
Open Access

Supporting Information

for *Adv. Sci.*, DOI 10.1002/advs.202304318

An Intelligent Synthetic Bacterium for Chronological Toxicant Detection, Biodegradation, and Its Subsequent Suicide

*Huan Liu, Lige Zhang, Weiwei Wang, Haiyang Hu, Xingyu Ouyang, Ping Xu and Hongzhi Tang**

Supplementary Information

An intelligent synthetic bacterium for chronological toxicant detection, biodegradation, and its subsequent suicide

Huan Liu†, Lige Zhang†, Weiwei Wang, Haiyang Hu, Xingyu Ouyang, Ping Xu, and Hongzhi Tang*

State Key Laboratory of Microbial Metabolism, Joint International Research Laboratory of Metabolic and Developmental Sciences, and School of Life Sciences and Biotechnology, Shanghai Jiao Tong University, Shanghai, People's Republic of China

†These authors contributed equally to this study.

*Corresponding author: H. Z. Tang

Mailing address: School of Life Sciences & Biotechnology, Shanghai Jiao Tong University, Shanghai 200240, P. R. China

Email: tanghongzhi@sjtu.edu.cn, Tel: +86-21-34204066, Fax: +86-21-34206723

Supplementary Table 1. List of plasmids used in this study.

Plasmid	Description	Reference
pS8K	BioBrick vector, pSC101 ori, Kan^R	[1]
pA1a	BioBrick vector, p15A ori, Amp^R	
J61002	BioBrick vector, pMB1 ori, Amp^R	iGEM*
pSB1C3	BioBrick vector, pMB1 ori, Cam^R	iGEM*
pSB1C3-WT	pSB1C3 carrying T-*nahR*-34-P_{sal}-*mrfp*-T	This study
pSB1C3-100-30	pSB1C3 carrying T-*nahR*-34-J23100-P_{sal}-30-*mrfp*-T	This study
pSB1C3-100-31	pSB1C3 carrying T-*nahR*-34-J23100-P_{sal}-31-*mrfp*-T	This study
pSB1C3-100-32	pSB1C3 carrying T-*nahR*-34-J23100-P_{sal}-32-*mrfp*-T	This study
pSB1C3-100-35	pSB1C3 carrying T-*nahR*-34-J23100-P_{sal}-35-*mrfp*-T	This study
pSB1C3-111-30	pSB1C3 carrying T-*nahR*-34-J23111-P_{sal}-30-*mrfp*-T	This study
pSB1C3-111-31	pSB1C3 carrying T-*nahR*-34-J23111-P_{sal}-31-*mrfp*-T	This study
pSB1C3-111-32	pSB1C3 carrying T-*nahR*-34-J23111-P_{sal}-32-*mrfp*-T	This study
pSB1C3-105-30	pSB1C3 carrying T-*nahR*-34-J23105-P_{sal}-30-*mrfp*-T	This study
pSB1C3-105-31	pSB1C3 carrying T-*nahR*-34-J23105-P_{sal}-31-*mrfp*-T	This study
pSB1C3-105-32	pSB1C3 carrying T-*nahR*-34-J23105-P_{sal}-32-*mrfp*-T	This study
J61002-100-*mrfp*	J61002 carrying J23100-RBS-*mrfp*-T	This study
J61002-P_{katE}	J61002 carrying P_{katE}	This study
J61002-P_{fic}	J61002 carrying P_{fic}	This study
J61002-P_{csiE}	J61002 carrying P_{csiE}	This study
J61002-P_{bolA}	J61002 carrying P_{bolA}	This study
J61002-P_{osmY}	J61002 carrying P_{osmY}	This study
pA1a-P_{bolA}- *nagAaGHAb*	pA1a carrying P_{bolA}-*nagAaGHAb*-T, Δ *lacIq*-P_{trc}-RBS-*mrfp*	This study
pA1a-P_{fic}- *nagAaGHAb*	pA1a carrying P_{bolA}- *nagAaGHAb* -T, Δ *lacIq*-P_{trc}-RBS-*mrfp*	This study
pS8K-*hokD*	pS8K carrying P_{ara}-RBS-*hokD*-T, Δ *mrfp*	This study
pS8K-*mazF*	pS8K carrying P_{ara}-RBS-*mazF*-T, Δ *mrfp*	This study
pS8K-*relK*	pS8K carrying P_{ara}-RBS-*relK*-T, Δ *mrfp*	This study
pS8K-*proE*	pS8K carrying P_{ara}-RBS-*proE*-T, Δ *mrfp*	This study

Plasmid	Description	Reference
pS8K-*gp2*	pS8K carrying P_{ara}-RBS-*gp2*-T, Δ *mrfp*	This study
pS8K-*ccdB*(L45)	pS8K carrying P_{ara}-RBS-*ccdB*-*dnaE*(L42)-T, Δ *mrfp*	This study
pS8K-*ccdB*(V46)	pS8K carrying P_{ara}-RBS-*ccdB*-*dnaE*(V46)-T, Δ *mrfp*	This study
pS8K-*ccdB*(K45)	pS8K carrying P_{ara}-RBS-*ccdB*-*dnaE*(K45)-T, Δ *mrfp*	This study
pS8K-*ccdB*(R40)	pS8K carrying P_{ara}-RBS-*ccdB*-*dnaE*(R40)-T, Δ *mrfp*	This study
pS8K-*nucB*	pS8K carrying P_{ara}-RBS-*nucB*-T, Δ *mrfp*	This study
pS8K-Converter *nucB*	pS8K carrying T-*nahR*-P_{sal}-*cI*-P_R-30-*nucB*-T, Δ *araC*-P_{ara}-RBS-*mrfp*	This study
pS8K-Converter *ccdB*	pS8K carrying T-*nahR*-P_{sal}-*cI*-P_R-30-*ccdB*-*dnaE*(L42)-T, Δ *araC*-P_{ara}-RBS-*mrfp*	This study
pS8K-Converter *(mrfp-ccdB)*	pS8K carrying T-*nahR*-P_{sal}-*cI*-P_R-30-*mrfp*-*ccdB*-*dnaE*(L42)-T, Δ *araC*-P_{ara}-RBS	This study
pS8K-Converter (*ccdB*-*mrfp)*	pS8K carrying T-*nahR*-P_{sal}-*cI*-P_R-30-*ccdB*-*dnaE*(L42)-*mrfp*-T, Δ *araC*-P_{ara}-RBS	This study
pS8K-toxin/antitoxin	pS8K carrying T-*ccdB*-*dnaE*(L42)-*nahR*-P_{sal}-30-*ccdA*-T, Δ *araC*-P_{ara}-RBS-*mrfp*	This study
pS8K-toxin/antitoxin(2)	pS8K carrying T-*ccdB*-*dnaE*(L42)-*nahR*-P_{sal}-32-*ccdA*-T, Δ *araC*-P_{ara}-RBS-*mrfp*	This study
pS8K-toxin/antitoxin(2)-*mrfp*	pS8K carrying T-*ccdB*-*dnaE*(L42)-*nahR*-P_{sal}-32-*ccdA*-RBS3-*mrfp*-T, Δ *araC*-P_{ara}-RBS	This study

iGEM* means this plasmid is from iGEM registry (http://parts.igem.org/Main_Page). "100", "30" and "32" respectively represent the registry numbers of promoter (J23100) and RBSs (B0030, B0032) from iGEM.

Supplementary Table 2. List of parts used in this study.

Part Name	Description and Source	DNA sequence (5'-3)
nahR	An LysR-type transcriptional activator of the ***nah*** and ***sal*** promoters.[2]	atggaactgcgtgacctggatttaaacctgctggtggtgttcaaccagttgctggtcg<br>acagacgcgtctctatcactgcggagaacctgggcctgacccagcctgccgtgagc<br>aatgcgctgaaacgcctgcgcacctcgctacaggacccactcttcgtgcgcacaca<br>tcagggaatggaacccacaccctatgccgcgcatctggccgagcccgtcacttcgg<br>ccatgcacgcactgcgcaacgccctacagcaccatgaaagcttcgatccgctgacc<br>agcgagcgtaccttcaccctggccatgaccgacattggcgagatctacttcatgccg<br>cggctgatggatgtgctggctcaccaggcccccaattgcgtgatcagtacggtgcg<br>cgacagttcgatgagcctgatgcaggccttgcagaacggaaccgtggacttggccg<br>tgggcctgcttcccaatctgcaaactggcttctttcagcgccggctgctccagaatca<br>ctacgtgtgcctatgtcgcaaggaccatccagtcacccgcgaacccctgactctgga<br>gcgcttctgttcctacggccacgtgcgtgtcatcgccgctggcaccggccacggcg<br>aggtggacacgtacatgacacgggtcggcatccggcgcgacatccgtctggaagt<br>gccgcacttcgccgccgttggccacatcctccagcgcaccgatctgctcgccactgt<br>gccgatacgtttagccgactgctgcgtggagcccttcggcctaagcgccttgccgca<br>cccagtcgtcttgcctgaaatagccatcaacatgttctggcatgcgaagtaccacaag<br>gacctagccaatatttggttgcggcaactgatgtttgacctgtttacggattga
nagAaG-HAb	Salicylate 5-hydroxylase (S5H)[3]	atggaactggtagtagaacccctcaatttgcatctgaacgcggagaccggcagcac<br>cctgcttgacgtgctcaggtccaacgaggtccccatttcttatagctgcatgtcgggc<br>cgctgcggcacttgccgttgccgtgtgattgccggccatcttcgcgataacggcccc<br>gagacagggcgcccgcaggcaggaaagggaacctacgttctggcctgtcaggcg<br>gttctgaccgaagactgcacgatcgagattcctgaatctgacgagatcgtggttcacc<br>cggcgcgcatcgtcaaggggacggtcacagcgatagacgaagccacccatgaca<br>tccggcgcctgcgcatcaaactggccaaaccgcttgagttcagccctggccagtac<br>gcaacggtgcagttcacgcccgaatgcgtccgcccatattcgatggccgggctgcc<br>tagcgatgcggaaatggagtttcagattcgcgcggttccgggcgggcatgtcagca<br>actacgttttcaatgaactgtccgtaggcgcttcggtgcggatcagcggccccctcg<br>gaacggcttatctgcggcgcacgcacaccggccccatgctttgtgtgggggtgga<br>acaggtctggcgcccgtcctttcgatcgttcgaggcgcactggaaagcgggatgag<br>caaccccatccatctgtacttcggtgtgcggagcgagcaggacatctatgacgagg<br>aacgccttcacgcattggctgcaaggtttccgaatctcaaggtgaatgtcgttgttgca<br>acaggccctgccggccctggtcgtcgatccggcctggtcaccgatctgatcggccg<br>tgacttgcccaatttggcgggatggcgcgcctacctgtgtggcgctccggccatggt<br>cgaggccctgaacctgctcgttgctcgcctaggcatagtacccgggcacatccatg<br>ccgatgcgttctatcccagcggcgtctgagcgaaggcaccatgcgaacccaattca<br>acccaaggataccaagccatgagtgaaccccaacgattaaaacccgtgtttcccca<br>agatccgaaatggccgggcgaaggtagcagccgcgttcccttctgggcctacacc<br>cgcgaagacctgtacaagcgcgaattggagcgcctgttctatgcaaaccactggtg<br>ctatgtaggcctggaagccgagattccgaatccaggcgacttcaagcgaacggtga<br>tcggtgagcgctcggtcatcatggtgcgtgatccggatggcggcatcaacgtggtg

Part Name	Description and Source	DNA sequence (5’-3)
nagAaG-HAb	Salicylate 5-hydroxylase (S5H)[3]	gagaacgtctgcgcccaccgtggcatgcgcttttgccgcgagcgccacggcaacg<br>ccaaggacttcttctgcccctaccaccagtggaactacagcctcaagggtgacctgc<br>agggcgtgcccttccgccgtggcgtcaagcaggacggcaaggtcaacggcggca<br>tgcccaaggacttcaaactcgaagaacacggcctgaccaagctcaaggtggccgc<br>ccgaggcggtgcagtgtttgcctcttttgaccacgatgtcgagcccttcgaggagttc<br>ctgggcccaaccatcctgcattacttcgaccgcgtcttcaacggccgcaagctcaag<br>atcctgggctaccgccgccagcgcatcccgggcaactggaagctgatgcaggaga<br>acatcaaggacccctaccacccgggcctgctgcacacctggttctcgaccttcggg<br>ctctggcgcgccgacaacaagtcggaactgaagatggacgccaagttccgccacg<br>ccgcaatgatctccacgcgcggtcagggcggcaagaacgaggaggtcgtgtccg<br>gcgtggacagcttcaaggaacagatgaaggtgaacgacccgcgcctgctcgacat<br>cgtgcccgagccctggtggggcggtccgactgcggtgatgaccacgatcttcccca<br>gcgtgatcatccagcagcaggtcaacagcgtatcgacccgccacatccagcccaa<br>cggtcacggctccttcgatttcgtctggacccacttcggcttcgaggacgacaacga<br>ggagtggacccagcgccgcctgatccaggccaacctgttcgggccggcgggcttc<br>gtgtcggccgatgacggcgaggtgatcgagtggtcgcaggaaggctttgagcaaa<br>aaccgacgcaccgcaccgtgatcgagatgggcggtcacgaaatcggcgacacgg<br>accacatggtcaccgagacgctgatccgcggcatgtacgactactggcgcaaggt<br>gatgggggaataaacatggtcgacttcaaaacctatttcgaactgctgaacctgtaca<br>gcgactacgccatggtgtgcgactccgccaattgggagaagtggcctgatttcttcat<br>cgagaccggcacctaccgcctgcagccgcgcgagaactttgagcagggcctgcc<br>gctgtgcctgctggcgctggagagcaaggcaatgatccgcgaccgggtgtacggc<br>gtcaaggaaaccatgtaccacgacccctactaccagcgccacatcgtgggcacgc<br>cgcgcgtgctgtcagtggagcgtgacgcggatggcgagcgcatcaccgccgaag<br>ccagctatgccgtgattcgcaccaagtacgacggcgattccacgattttcaacgccg<br>gctattaccgagacgtgatcgtgcgcacgcccgagggcctcaagctgaagtcgcg<br>cctgtgcgtttacgacagcgaaatgatccccaactctgtgatctatccaatctgaggc<br>gacgcacatgactcagaactggattgatgcagcttgtcttgacgacatccctgaagg<br>cgatgtggtcggcgtcaaagttaacggcaaggaaattgcgctctacgaggtcgagg<br>gggagatttatgccaccgataacctgtgcacgcacggcgctgcgcgcatgagcgat<br>ggctttctcgaaggccgggaaattgaatgccctctgcaccaaggtcgatttgatgtct<br>gcacaggcaaagccttgtgcaccccccctgacaaaggacatcaaaacctaccccgtc<br>aaaattgaaaacatgcgcgtgatgctcaaaatggagtaa
cI repre-sssor	CI repressor binds to the CI regulator to suppress gene expression. BBa_C0051	atgagcacaaaaaagaaaccattaacacaagagcagcttgaggacgcacgtcgcc<br>ttaaagcaatttatgaaaaaaagaaaaatgaacttggcttatcccaggaatctgtcgca<br>gacaagatggggatggggcagtcaggcgttggtgctttatttaatggcatcaatgcat<br>taaatgcttataacgccgcattgcttgcaaaaattctcaaagttagcgttgaagaattta<br>gcccttcaatcgccagagaaatctacgagatgtatgaagcggttagtatgcagccgt<br>cacttagaagtgagtatgagtaccctgtttttctcatgttcaggcagggatgttctcac<br>ctgagcttagaacctttaccaaaggtgatgcggagagatgggtaagcacaaccaaa<br>aaagccagtgattctgcattctggcttgaggttgaaggtaattccatgaccgcaccaa<br>caggctccaagccgagctttcctgacggaatgttaattctcgttgaccctgagcaggc

Part Name	Description and Source	DNA sequence (5’-3)
cI repre-sssor	CI repressor binds to the CI regulator to suppress gene expression. BBa_C0051	tgttgagccaggtgatttctgcatagccagacttgggggtgatgagtttaccttcaaga<br>aactgatcagggatagcggtcaggtgtttttacaaccactaaacccacagtacccaat<br>gatcccatgcaatgagagttgttccgttgtggggaaagttatcgctagtcagtggcct<br>gaagagacgtttggcgctgcaaacgacgaaaactacgctttagtagcttaataacgc<br>tgatagtgctagtgtagatcgc
ccdB	Toxin that poisons and kills *E. coli* by inhibiting the DNA gyrase and inducing double strand breaks. BBa_K1318000	atgcagttcaaggtgtacacctacaagcgtgagtcgcgttaccgtctgttcgtcgatgt<br>gcagtcggacatcatcgacacgcccggccgtcgtatggtcatcccgctggccagc<br>gcccgtctgctgtcggacaaggtcagccgcgagctgtacccggtggtgcacgtgg<br>gcgacgagagctggcgcatgatgaccaccgacatggcctcggtcccggtcagcgt<br>catcggcgaggaggtggccgacctgtcgcaccgtgagaacgacatcaagaacgc<br>catcaacctgatgttctggggcatctga
ccdA	CcdA inhibits the toxin CcdB by binding to it, thus represses cell death. BBa_K1075032	atgaaacagcgcattaccgtgaccgtggatagcgatagctatcagctgctgaaagc<br>gtatgatgtgaacattagcggcctggtgagcaccaccatgcagaacgaagcgcgtc<br>gcttacgcgcggaacgttggaaagcggaaaatcaggaaggcatggcggaagtgg<br>cgcgttttattgaaatgaacggcagctttgcggatgaaaaccgcgattggtaaaacg<br>cggtgtaaggcctgcatctg
C-*dnaE*	They are autocatalytically excised in a process called protein splicing, and allow the reconstitution of the mature protein.[4] It is linked to the N-terminal of split protein.	atgatcaagatcgccacccgtaagtacctgggcaagcagaacgtctacgacatcgg<br>cgtggagcgcgaccacaacttcgccctgaagaacggcttcatcgcctcgaac
N-*dnaE*	It is linked to the C-terminal of split protein.	tgcctgagctacgagaccgagatcctgaccgtggagtacggcctgctgccgatcgg<br>caagatcgtggagaagcgtatcgagtgcaccgtctactcggtggacaacaacggc<br>aacatctacacccagccggtcgcccagtggcacgaccgcggcgagcaggaggtc<br>ttcgagtactgcctggaggacggctcgctgatccgcgccaccaaggaccacaagtt<br>catgaccgtggacggccagatgctgccgatcgacgaaattttcgagcgtgagctgg<br>acctgatgcgtgtggacaacctgccgaactga
nucB	A biofilm-degrading endonuclease[5]	atgaaaaagtggatggcgggcctgtttctggcggcggcggttttattatgtctgatggt<br>tcctcaacagattcagggcgcgagcagctatgataaagtgctgtattttccgctgagc<br>cgctatccggaaaccggcagccatattcgcgatgcgattgcggaaggccatccgg<br>atatttgcaccattgatcgcgatggcgcggataaacgccgcgaagaaagcctgaaa<br>ggcattccgaccaaaccgggctatgatcgcgatgaatggccgatggcggtgtgcg<br>aagaaggcggcgcgggtgcggatgttcgttatgtgacccctagcgataaccgcgg<br>cgcgggcagctgggttggcaatcagatgtcaagctatccggatggcacccgcgtg<br>ctgtttattgtgcagtaa

Part Name	Description and Source	DNA sequence (5’-3)
hokD	It causes cell death by elimination of vital cell wall functions. BBa_K1497008	atgaagcagcaaaaggcgatgttaatcgccctgatcgtcatctgtttaaccgtcatagtgacggcactggtaacgaggaaagacctctgcgaggtacgaatccgaaccggccagacggaggtcgctgtcttcacagcttacgaacctgaggagtaa
gp2	It binds to the β’ subunit of RNA polymerase to inhibit host transcription. BBa_K1893019	atgagcaatgttaataccggctcactgtcagttgataataaaaaattttgggccaccgtggaatcaagcgaacatagttttgaagttccgatttatgcagaaaccttagatgaagcattagaactggccgaatggcagtatgtgccggcaggctttgaagtgacccgcgttcgtccgtgtgttgccccgaaataa
relK	It causes mRNA degradation in vivo resulting in significant changes to the proteome.[6]	gtgcgtagtgtgaattttgatccggatgcctgggaagattttctgttttggttagcagccgatcgtaaaaccgcacgtcgcattacccgcttaattggcgaaattcagcgcgatccgtttagtggtattggcaaaccggaaccgttacagggcgaactgagcggctattggagtcgccgtattgatgatgaacatcgtctggtgtatcgtgcaggtgatgatgaagtgaccatgctgaaagcacgctatcattattaa
proE	It induces lysis by formation of a transmembrane tunnel structure in the cell envelope of *E. coli*.[7]	atggtgcgttggaccctgtgggataccttagcatttctgctgctgttatcattattactgccgagtctgttaattatgtttattccgtctacctttaaacgtccggtgagtagctggaaagccctgaatctgcgcaaaaccttactgatggcctctagcgtgcgcctgaaaccgctgaattgtagtcgcttaccgtgtgtgtatgcacaggaaaccctgacctttctgctgacccagaaaaaaacctgtgttaaaaattatgttcgtaaagaataa
P_{sal}	Bidirectional promoter controlled by NahR.[2]	ggggcctcgcttgggttattgctggtgcccggccgggcgcaatattcatgttgatgatttattatatatcgagtggtgtatttatcaatattgtttgctccgttatcgttattaacaagtcatcaataaagccatcacgagtaccatag
P_R	The promoter has two DNA binding sites for lambda CI repressor. BBa_R0051	taacaccgtgcgtgttgactattttacctctggcggtgataatggttgc
P_{osmY}	Stationary phase Promoter BBa_J45992	ctggcacaggaacgttatccggacgttcagttccaccagacccgcgagcattaattcttgcctccagggcgcggtagtggcgccctgtcaatttcccttccttattagccgcttacggaatgttcttaaaacattcacttttgcttatgttttcgctgatatcccgagcggtttcaaaattgtgatctatatttaacaa
P_{katE}	Stationary phase promoter[8]	gcagaaatgactctcccatcagtacaaacgcaacatatttgccacgcagcatccagacatcacgaaacgaatccatctttatcgcatgttctggcggcgcgggttccgtgcgtgggacatagctaataatctggcggttttgctggcggagcggtttcttcattactggcttcactaaacgcatattaaaaatcagaaaaactgtagtttagccgatttagcccctgtacgtcccgc

Part Name	Description and Source	DNA sequence (5'-3)
P_{csiE}	Stationary phase promoter[8]	tgtttatcgcgaacagatggtggactttatccttagcgcgttgaatccgcagaactaacccatgatcgctagcacgataatcattcacaaaaccaccttaagacatgctaatccactggtcagaacagtttaagatgagaaaaattctgtgacgcttgccaacatttctgatgattagcattcccttcgtcatttccctgagcaaactttagctattcttatcaattatgcttatgggagatctacag
P_{bolA}	Stationary phase promoter[8]	tgtttggtaaaaattcccgccatcataacattgccaacggcgaggggaagtgggtaaggcatgtaaattcatcatgttggcgaaataatcgcccctggtaaaagaaacactgatgcgaggcctgtgtttcaatctttaaatcagtaaacttcatacgcttgacggaaaaaccaggacgaaacctaaatatttgttgttaagctgcaatggaaacggtaaaagcggctagtatttaaag
P_{fic}	Stationary phase promoter[8]	ttgaccatggtcagcgtgatttcggttacgcggtatttggtaaagtggtgaaaggcatggacgttgccgataagatttcccaggtgccgactcatgacgttggtccgtaccagaatgtgccgtcaaaaccggtagttatcctttccgctaaagtcctgccgtaatgatttctcgcgcgggcaatcttgcccgcgcttctgctCtccCggcgtaacccggatttgccgcttatacttgtggcaaatggacacgttcagggaggcatcaagtgaagaaactcaccgataagcaaaagtcccgtctctgggagcttcagcgtaatcgtaattttcaggccagtcgccgtcttgaaggcgtcgagatgcctttagtcactcttactgccgcagaggctttagcgcgccttgaagagctgaggagtcactatgagcgataaattcggcgaagggcgcgatccgtatctttatccaggccttgat
J23100	Constitutive promoter BBa_J23100	ttgacggctagctcagtcctaggtacagtgctagc
J23111	Constitutive promoter BBa_J23111	ttgacggctagctcagtcctaggtatagtgctagc
J23105	Constitutive promoter BBa_J23105	atttacggctagctcagtcctaggtactatgctagc
30	RBS BBa_B0030	attaaagaggagaaa
31	RBS BBa_B0031	tcacacaggaaacc
32	RBS BBa_B0032	tcacacaggaaag
34	RBS BBa_B0034	aaagaggagaaa
35	RBS BBa_B0035	tctagagattaaagaggagaatactag
RBS1	RBS from plasmid pA1a	tttaagaaggagatatacat

Part Name	Description and Source	DNA sequence (5'-3)
B0015	Double terminator BBa_B0015	ccaggcatcaaataaaacgaaaggctcagtcgaaagactgggcctttcgttttatctgttgtttgtcggtgaacgctctctactagagtcacactggctcaccttcgggtgggcctttctgcgtttata

"BBa_" represents a part from iGEM registry (http://parts.igem.org/Main_Page).

Supplementary Table 3. List of primers used in this study.

Primer Name	DNA Sequence (5'-3')	Usage
pSB1C3 F	TACTAGTAGCGGCCGCTGCAGTCCG	Construction and optimization of salicylic acid biosensor
pSB1C3 R	CTCTAGAAGCGGCCGCGAATTCCAG	
nahR + P_{sal} F	CGGCCGCTTCTAGAGTCAATCCGTAAACAGGTCAAACATC	
nahR + P_{sal} R	TTCGGAGGAAGCCATTTTCTCCTCTTTAATCTATGGTACTCG	
mrfp F	ATTAAAGAGGAGAAAATGGCTTCCTCCGAAGACGT	
mrfp R	CGGCCGCTACTAGTATTAAGCACCGGTGGAGTGAC	
J23100-B0032 F	AGTCACACAGGAAAGATGGCTTCCTCCGAAGAC	
J23100-B0032 R	CTTTCCTGTGTGACTATGGTACTCGTGATGGCTT	
J23100-B0031 F	AGTCACACAGGAAACCATGGCTTCCTCCGAAGAC	
J23100-B0031 R	GGTTTCCTGTGTGACTATGGTACTCGTGATGGCTT	
J23111-P_{sal} F	GCTAGCACTATACCTAGGACTGAGCTAGCCGTCAAGGGGCCTCGCTTGGGTTA	
J23111-B0030 R	TTTCTCCTCTTTAATCTATGGTACTCGTGATGGCTT	
J23111-B0030 F	AGATTAAAGAGGAGAAAATGGCTTCCTCCGAAGAC	
SAbio (-P_{sal}, -J23111) R	AGGTATAGTGCTAGCAAAGAGGAGAAAATGGAACTGCG	
J23105-P_{sal} F	GCTAGCATAGTACCTAGGACTGAGCTAGCCGTAAATGGGGCCTCGCTTGGGTTA	
SAbio (-P_{sal}, -J23105) R	AGGTACTATGCTAGCAAAGAGGAGAAAATGGAACTGCG	
J61002 F	GAATTCCAGAAATCATCCTTAGCG	Selection of stationary phase promoters
J61002 R	TCTAGAGTACTAGTGAAAGAGGAGAAA	
P_{osmY}-J61 F	AAGGATGATTTCTGGAATTCCTGGCACAGGAACGTTATC	
P_{osmY}-J61 R	CCTCTTTCACTAGTACTCTAGATTGTTAAATATAGATCACAATTTTGAAACCGC	

Primer Name	DNA Sequence (5’-3’)	Usage
P_{csiE}-J61 F	AAGGATGATTTCTGGAATTCTGTTTATCGCGAACAGATGG	Selection of stationary phase promoters
P_{csiE}-J61 R	CCTCTTTCACTAGTACTCTAGACTGTAGATCTCCCATAAGCAT	
P_{bolA}-J61 F	AAGGATGATTTCTGGAATTCTGTTTGGTAAAAATTCCCGC	
P_{bolA}-J61 R	CCTCTTTCACTAGTACTCTAGACTTTAAATACTAGCCGCTTT	
P_{katE}-J61 F	AAGGATGATTTCTGGAATTCGCAGAAATGACTCTCCCATC	
P_{katE}-J61 R	CCTCTTTCACTAGTACTCTAGAGCGGGACGTACAGGGGCTAA	
P_{fic}-J61 F	AAGGATGATTTCTGGAATTCTTGACCATGGTCAGCGTG	
P_{fic}-J61 R	CCTCTTTCACTAGTACTCTAGAATCAAGGCCAGGATAAAGAT	
P_{ara}-J61 F	AAGGATGATTTCTGGAATTCTTATGACAACTTGACGGCTA	
P_{ara}-J61 R	CCTCTTTCACTAGTACTCTAGAATGGAGAAACAGTAGAGAG	
J23100-J61 F	TTGACGGCTAGCTCAGTCCTAGGTACAGTGCTAGCGAATTCCAGAAATCATCCTTAGCG	
J23100-J61 R	GCTAGCACTGTACCTAGGACTGAGCTAGCCGTCAATCTAGAGTACTAGTGAAAGAGGAGAAA	
pA1a F	GGATCCAAACTCGAGTAAGGATCT	Construction of biodegradation module
pA1a R	ATTCACCACCCTGAATTGACTCTC	
P_{bol} (-pA, -S5H) F	TTCAGGGTGGTGAATTGTTTGGTAAAAATTCCCGC	
P_{bol} (-pA, -S5H) R	TACTACCAGTTCCATCTTTAAATACTAGCCGCTTT	
P_{fic} (-pA, -S5H) F	TTCAGGGTGGTGAATTTGACCATGGTCAGCGTGAT	
P_{fic} (-pA, -S5H) R	TACTACCAGTTCCATATCAAGGCCTGGATAAAGATACG	
S5H (-P_{bol}) F	GGCTAGTATTTAAAGATGGAACTGGTAGTAGAACC	
S5H (-P_{fic}) F	TATCCAGGCCTTGATATGGAACTGGTAGTAGAACC	
S5H(-pA) R	CTCGAGTTTGGATCCTTACTCCATTTTGAGCATCAC	

Primer Name	DNA Sequence (5'-3')	Usage
pS8K (-RFP) F	GGATCCAAACTCGAGTAAGG	Selection of toxic proteins
pS8K (-RFP) R	ATGTATATCTCCTTCTTAAAAGATC	
gp2-pS8K F	GAAGGAGATATACATATGAGCAATGTTAATACCGG	
gp2-pS8K R	CTCGAGTTTGGATCCTTATTTCGGGGCAACACA	
hokD-pS8K F	GAAGGAGATATACATATGAAGCAGCAAAAGGC	
hokD-pS8K R	CTCGAGTTTGGATCCTTACTCCTCAGGTTCGTAAG	
mazF-pS8K F	GAAGGAGATATACATATGGTAAGCCGATACGTACC	
mazF-pS8K R	CTCGAGTTTGGATCCCTACCCAATCAGTACGTTAA	
proE-pS8K F	GAAGGAGATATACATATGGTGCGTTGGACC	
proE-pS8K R	CTCGAGTTTGGATCCTTATTCTTTACGAACATAATTTTTAACA	
relK-pS8K F	GAAGGAGATATACATGTGCGTAGTGTGAATTTTGA	
relK-pS8K R	CTCGAGTTTGGATCCTTAATAATGATAGCGTGCTTTCA	
nucB-pS8K F	GAAGGAGATATACATATGAAAAAGTGGATGGCGGG	
nucB-pS8K R	CTCGAGTTTGGATCCTTACTGCACAATAAACAGCACG	
ccdB-pS8K F	GAAGGAGATATACATATGCAGTTCAAGGTGTACAC	
ccdB-pS8K R	CTCGAGTTTGGATCCTCAGATGCCCCAGAACATCA	
N-*ccdB*-L42 R	CTCGTAGCTCAGGCACAGCAGACGGGCGCT	
N-*ccdB*-V46 R	CTCGTAGCTCAGGCAGACCTTGTCCGACAGCAGA	
N-*ccdB*-R40 R	CTCGTAGCTCAGGCAACGGGCGCTGGCCAG	
N-*ccdB*-K45 R	CTCGTAGCTCAGGCACTTGTCCGACAGCAGACG	
dnaE-L42 F	AGCGCCCGTCTGCTGTGCCTGAGCTACGAGACCGA	
dnaE-L42 R	GCTGACCTTGTCCGAGTTCGAGGCGATGAAGCC	

Primer Name	DNA Sequence (5'-3')	Usage
dnaE-V46 F	CTGTCGGACAAGGTCTGCCTGAGCTACGAGACCGA	Selection of toxic proteins
dnaE-V46 R	GTACAGCTCGCGGCTGTTCGAGGCGATGAAGCC	
dnaE-R40 F	CTGGCCAGCGCCCGTTGCCTGAGCTACGAGACCGA	
dnaE-R40 R	CTTGTCCGACAGCAGGTTCGAGGCGATGAAGCC	
dnaE-K45 F	CTGCTGTCGGACAAGTGCCTGAGCTACGAGACCGA	
dnaE-K45 R	CAGCTCGCGGCTGACGTTCGAGGCGATGAAGCC	
ccdB-C-L42 F	TTCATCGCCTCGAACTCGGACAAGGTCAGCCG	
ccdB-C-V46 F	TTCATCGCCTCGAACAGCCGCGAGCTGTACCC	
ccdB-C-R40 F	TTCATCGCCTCGAACCTGCTGTCGGACAAGGT	
ccdB-C-K45 F	TTCATCGCCTCGAACGTCAGCCGCGAGCTGTAC	
pS8K F	GGATCCAAACTCGAGTAAGGATCTC	Construction of converter suicide circuit
pS8K R	GACGTCGGAATTGCCAGCTGGG	
nahR (-pS8K) F	CAGCTGGCAATTCCGACGTCTCAATCCGTAAACAGGTCAAACATC	
nahR (-*cI*) R	TGGTTTCTTTTTTGTGCTCATTTTCTCCTCTTTAATCTATG	
cI (-*nahR*) F	CATAGATTAAAGAGGAGAAAATGAGCACAAAAAAGAAACCA	
cI (-*ccdB*) R	CATTTTCTCCTCTTTAATATTATTTCTAGAGCAACCATTATCAC	
ccdB (-*cI*) F	TGCTCTAGAAATAATATTAAAGAGGAGAAAATGCAGTTCAAGGTGTACAC	
nucB (-*cI*) F	TGCTCTAGAAATAATATTAAAGAGGAGAAAATGAAAAAGTGGATGGCGG	
mrfp (-*cI*) F	TGCTCTAGAAATAATTTTAAGAAGGAGATATACATATGGCGAG	
mrfp (-*ccdB*) R	CATTTTCTCCTCTTTAATTTAAGCACCGGTGGAGTGAC	
cI R	ATTATTTCTAGAGCAACCATTATCAC	

**Primer Name**	**DNA Sequence (5'-3')**	**Usage**
ccdB (-*mrfp*) F	TCCACCGGTGCTTAAATTAAAGAGGAGAAAATGCAGTTCAAGGTGTACAC	
mrfp (-*ccdB*) F	TTCTGGGGCATCTGATTTAAGAAGGAGATATACATATGGCGAG	
mrfp (-pS8K) R	CTCGAGTTTGGATCCTTAAGCACCGGTGGAGTGAC	
ccdB (-pS8K) F	GGCAATTCCGACGTCTCAGATGCCCCAGAACATCA	Construction of toxin/antitoxin suicide circuit
ccdB (-*nahR*) R	CTGTTTACGGATTGATTTAAGAAGGAGATATACATATGCAGT	
nahR-P_{sal}-30 F	TCAATCCGTAAACAGGTCA	
nahR-P_{sal}-30 R	TTTCTCCTCTTTAATCTATGGTACT	
ccdA (-*nahR*) F	ATTAAAGAGGAGAAAATGAAACAGCGCATTACCGT	
ccdA (-pS8K) R	TCGAGTTTGGATCCCCAGATGCAGGCCTTACACCG	
SAbio-B0035-*mrfp* F	TCTAGAGAAAGAGGAGAAATACTAGATGGCTTCCTCCGAAGAC	Optimization of the triple-plasmid transformant
SAbio-B0035-*mrfp* R	CTAGTATTTCTCCTCTTTCTCTAGACTATGGTACTCGTGATGGCT	
Toxin/antitoxin-B0032-*ccdA* F	TCTAGAGTCACACAGGAAAGTACTAGATGAAACAGCGCATTACCG	
Toxin/antitoxin-B0032-*ccdA* R	CTAGTACTTTCCTGTGTGACTCTAGACTATGGTACTCGTGATGGCT	
ccdA R	CAGATGCAGGCCTTACAC	
RBS-*mrfp* (-*ccdA*) F	TAAGGCCTGCATCTGTTTAAGAAGGAGATATACATATGGCG	

The underlined part is the homologous sequence for seamless cloning.

Supplementary Table 4. Amino acid sequences of the complexes of split CcdB and DnaE used for prediction of the complex structures.

Name of complex	Amino acid sequence
CcdB-C-R40-C-DnaE	MIKIATRKYLGKQNVYDIGVERDHNFALKNGFIASNLLSDKVS RELYPVVHVGDESWRMMTTDMASVPVSVIGEEVADLSHREND IKNAINLMFWGI
N-DnaE-L40-CcdB-N	MQFKVYTYKRESRYRLFVDVQSDIIDTPGRRMVIPLASARCLS YETEILTVEYGLLPIGKIVEKRIECTVYSVDNNGNIYTQPVAQW HDRGEQEVFEYCLEDGSLIRATKDHKFMTVDGQMLPIDEIFER ELDLMRVDNLPN
CcdB-C-L42-C-DnaE	MIKIATRKYLGKQNVYDIGVERDHNFALKNGFIASNSDKVSRE LYPVVHVGDESWRMMTTDMASVPVSVIGEEVADLSHRENDIK NAINLMFWGI
N-DnaE-S43-CcdB-N	MQFKVYTYKRESRYRLFVDVQSDIIDTPGRRMVIPLASARLLC LSYETEILTVEYGLLPIGKIVEKRIECTVYSVDNNGNIYTQPVAQ WHDRGEQEVFEYCLEDGSLIRATKDHKFMTVDGQMLPIDEIFE RELDLMRVDNLPN
CcdB-C-K45-C-DnaE	MIKIATRKYLGKQNVYDIGVERDHNFALKNGFIASNVSRELYP VVHVGDESWRMMTTDMASVPVSVIGEEVADLSHRENDIKNAI NLMFWGI
N-DnaE-V46-CcdB-N	MQFKVYTYKRESRYRLFVDVQSDIIDTPGRRMVIPLASARLLS DKCLSYETEILTVEYGLLPIGKIVEKRIECTVYSVDNNGNIYTQP VAQWHDRGEQEVFEYCLEDGSLIRATKDHKFMTVDGQMLPID EIFERELDLMRVDNLPN
CcdB-C-V46-C-DnaE	MIKIATRKYLGKQNVYDIGVERDHNFALKNGFIASNSRELYPV VHVGDESWRMMTTDMASVPVSVIGEEVADLSHRENDIKNAIN LMFWGI
N-DnaE-S47-CcdB-N	MQFKVYTYKRESRYRLFVDVQSDIIDTPGRRMVIPLASARLLS DKVCLSYETEILTVEYGLLPIGKIVEKRIECTVYSVDNNGNIYTQ PVAQWHDRGEQEVFEYCLEDGSLIRATKDHKFMTVDGQMLPI DEIFERELDLMRVDNLPN

‘CcdB-C’ and ‘C-DnaE’ represent the C-terminal part of split proteins, ‘CcdB-N’ and ‘N-DnaE’ represent the N-terminal part of split proteins. ‘R40, L42, K45, V46’ represent the different split sites of CcdB.

Supplementary Figure 1. Plasmid maps used in this study.

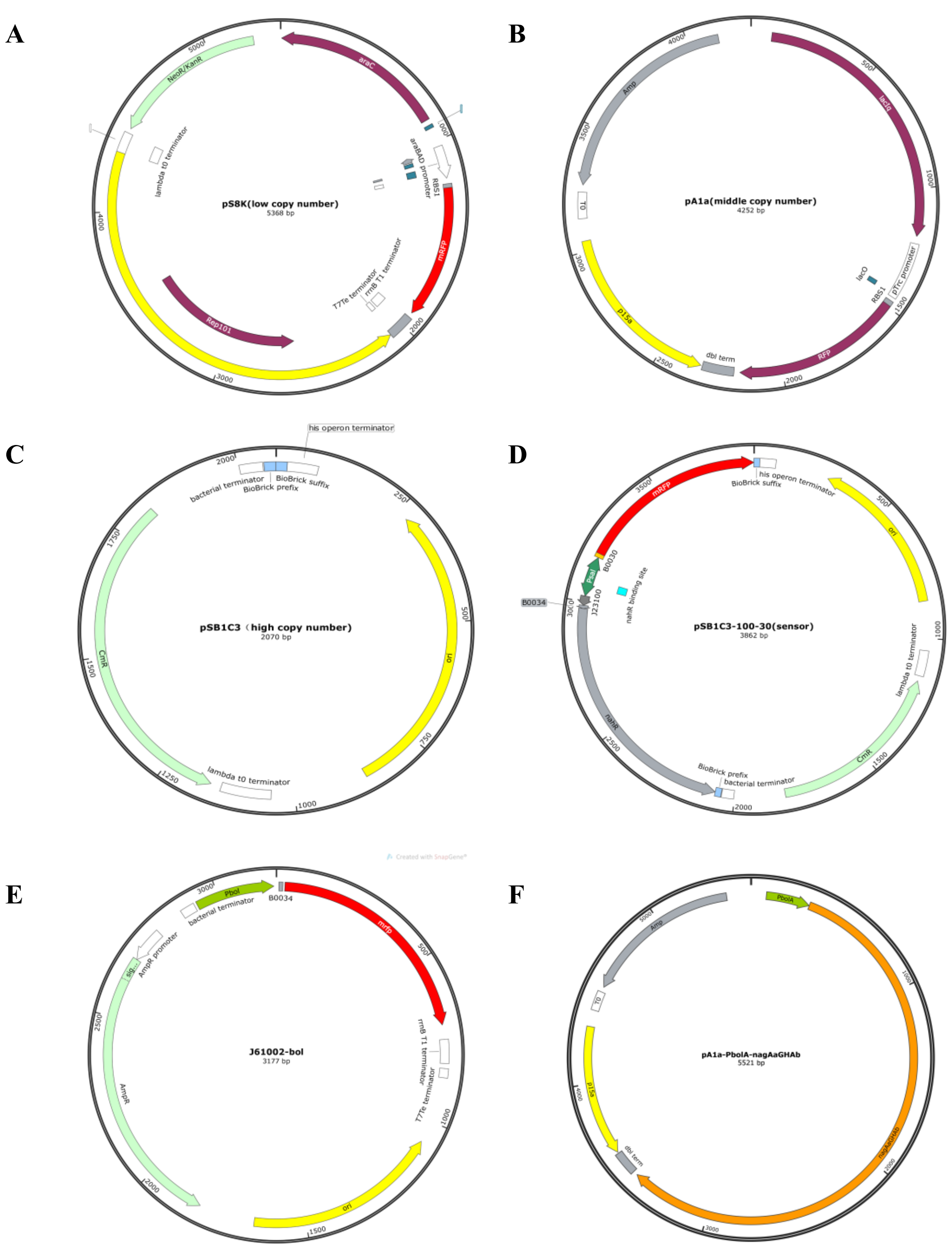

(See the next page)

(Follow the previous page)

G

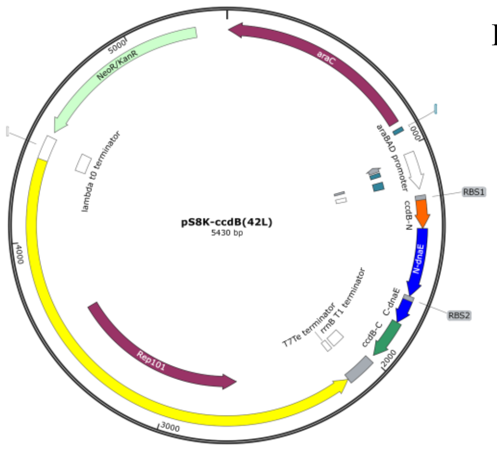

H

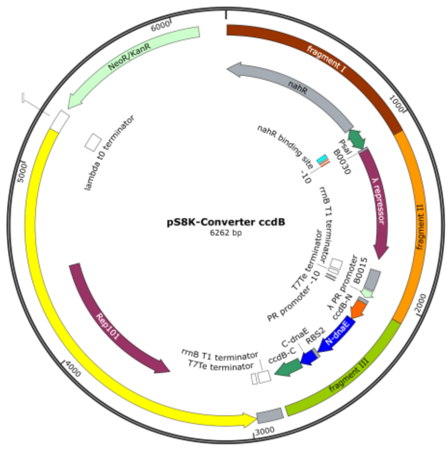

I

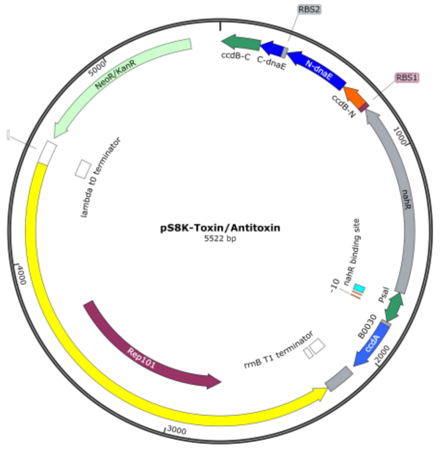

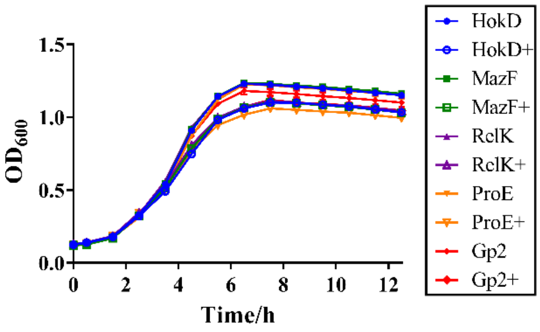

Supplementary Figure 2. Growth curves of strains containing different toxic proteins, and all functional toxic proteins are driven by an inducible promoter. “+” represents induction by arabinose. Values are mean ± s.d. (n = 3 biologically independent samples).

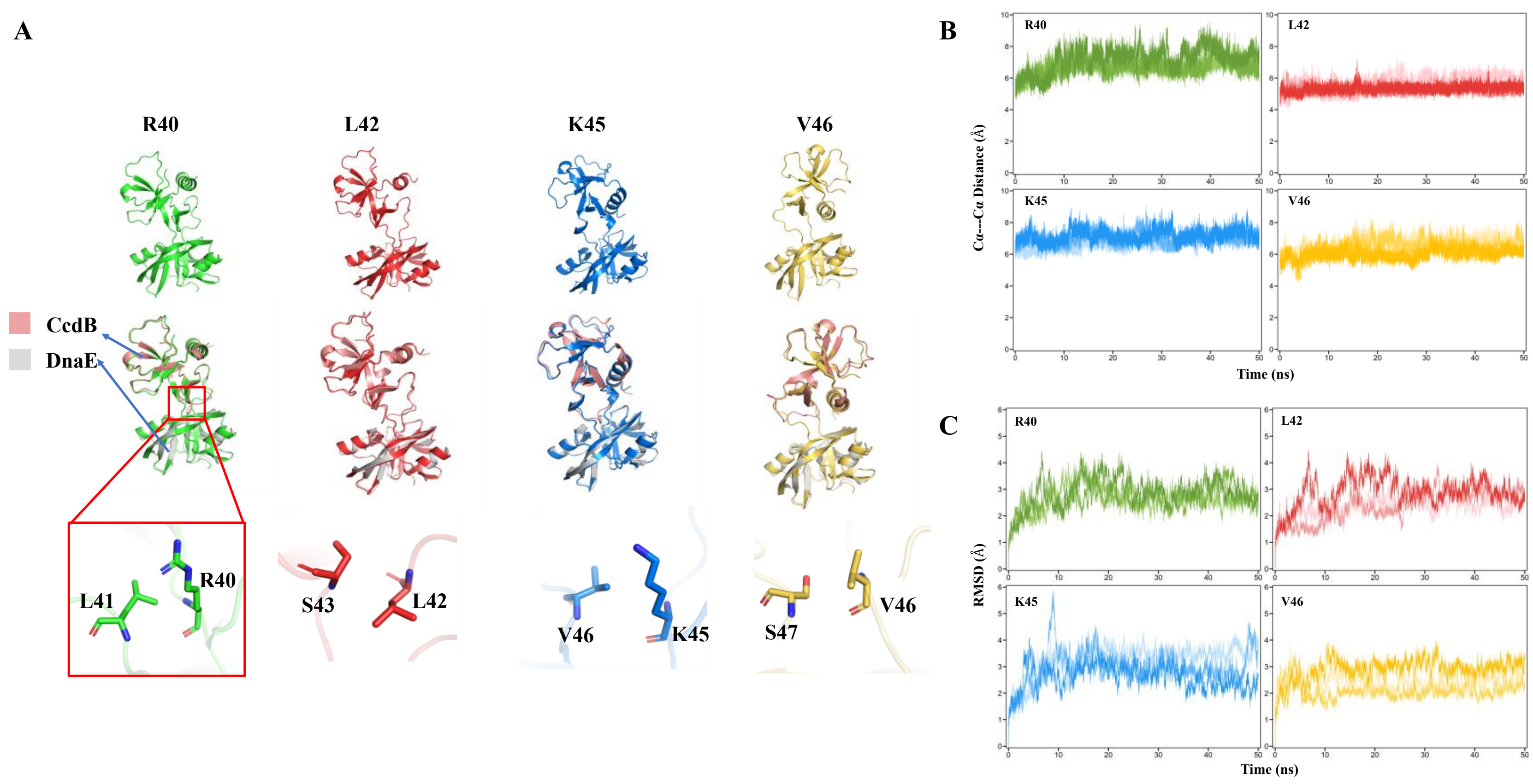

Supplementary Figure 3. Structure prediction and molecular dynamics (MD) simulation for the complexes of CcdB and DnaE with different split sites. **A**, Structure prediction of the complexes and alignment with original individual parts. **B**, In MD simulation sampling, the distribution of distances between the key residues C_α-C_α. **C**, In MD simulation sampling, the distribution of Root Mean Squared Error (RMSD) for the distances between key residues C_α-C_α.

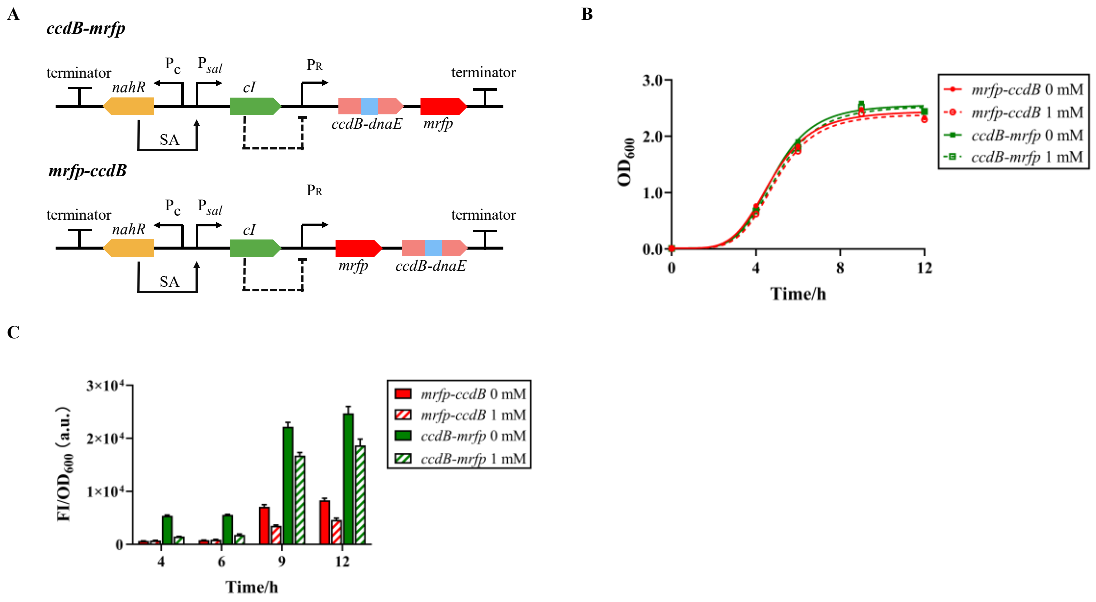

Supplementary Figure 4. mRFP was used to characterize the expression of CcdB in the "gene converter" circuit at different concentrations of SA. **A**, *mrfp* was introduced into different positions of the "gene converter" circuit **B,** Growth curves of strains containing 'gene converter' circuit with mRFP under different concentrations of SA. **C,** FI/$OD_{600\ nm}$ was used to represent the expression of CcdB under different concentrations of SA at different time. Values are mean ± s.d. ($n = 3$ biologically independent samples).

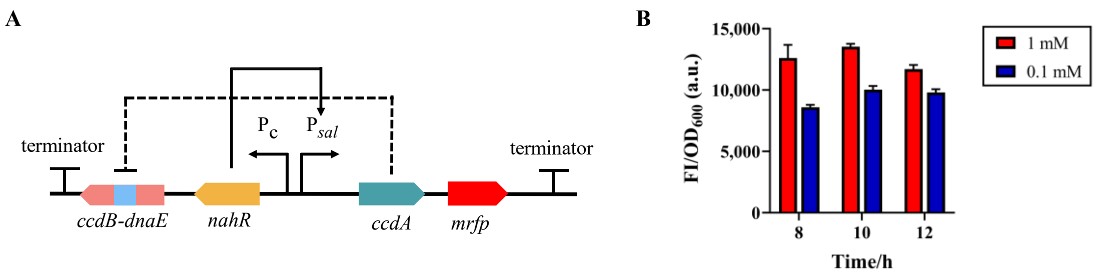

Supplementary Figure 5. mRFP was used to characterize the expression of CcdA in the double-plasmid transformant containing biodegradation and suicide circuit at 1- and 0.1- mM SA. Values are mean ± s.d. (*n* = 3 biologically independent samples).

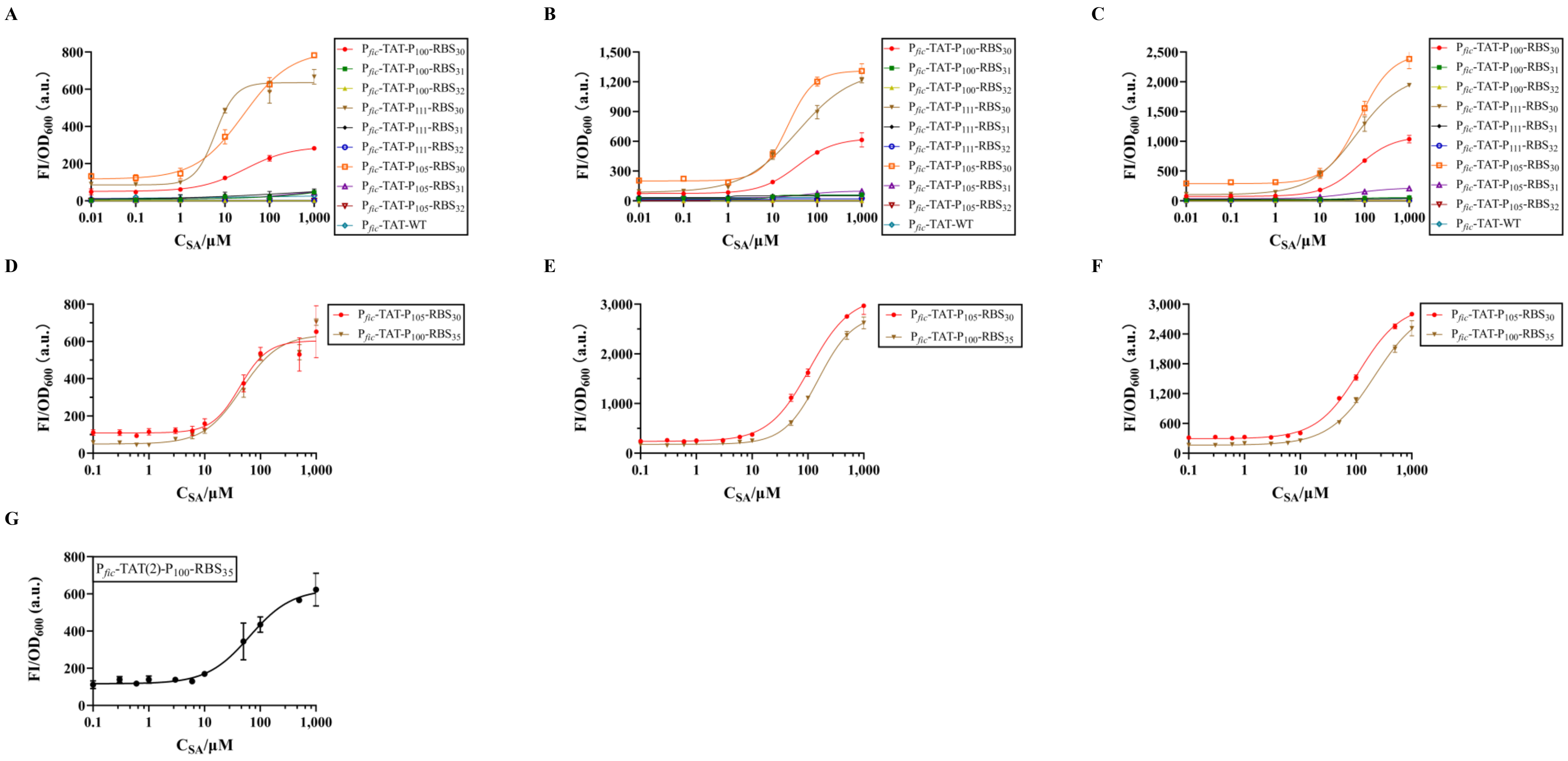

Supplementary Figure 6. Characterization of triple-plasmid transformants containing the biosensors, biodegradation module, and suicide circuit. **A–C**, Dose-Response curves of the triple-plasmid transformant with different optimized biosensors in 96-well plates, A–C are the results

for 6, 8, and 10 h. P_{fic}-TAT-P_{1XX}-RBS_{3X} indicates that the triple-plasmid transformant contains pSB1C3-1XX-3X (optimized biosensor), pA1a-P_{fic}-*nagAaGHAb* (biodegradation module) and pS8K-toxin/antitoxin (suicide circuit), WT is the wild-type biosensor. **D–F**, Dose-Response curves of the triple-plasmid transformant with different optimized biosensors in shake flasks. D–F are the results for 6, 8, and 10 h. **G,** Dose-Response curve of the triple-plasmid transformant with optimized biosensor and suicide circuit in shake flasks at 6 h. Values are mean ± s.d. (*n* = 3 biologically independent samples.

References:

[1] T. S. Lee, R. A. Krupa, F. Zhang, M. Hajimorad, W. J. Holtz, N. Prasad, S. K. Lee, J. D. Keasling, *J. Biol. Eng.* **2011**, 5, 12.

[2] Z. Huang, M. A. Schell, *J. Biol. Chem.* **1991**, 266, 10830.

[3] N. Y. Zhou, J. Al-Dulayymi, M. S. Baird, P. A. Williams, *J. Bacteriol.* **2002**, 184, 1547.

[4] R. Lopez-Igual, J. Bernal-Bayard, A. Rodriguez-Paton, J. M. Ghigo, D. Mazel, *Nat. Biotechnol.* **2019**, 37, 75.

[5] D. van Sinderen, R. Kiewiet, G. Venema, *Mol. Microbiol.* **1995**, 15, 213.

[6] S. B. Korch, V. Malhotra, H. Contreras, J. E. Clark-Curtiss, *J. Microbiol.* **2015**, 53, 783.

[7] A. Witte, G. Wanner, M. Sulzner, W. Lubitz, *Arch. Microbiol.* **1992**, 157, 381.

[8] G. Miksch, F. Bettenworth, K. Friehs, E. Flaschel, A. Saalbach, T. Twellmann, T. W. Nattkemper, *J. Biotechnol.* **2005**, 120, 25.
